# Accurate localization and coactivation profiles of the frontal eye field and inferior frontal junction: an ALE and MACM fMRI meta-analysis

**DOI:** 10.1007/s00429-023-02641-y

**Published:** 2023-04-24

**Authors:** Marco Bedini, Emanuele Olivetti, Paolo Avesani, Daniel Baldauf

**Affiliations:** 1 Center for Mind/Brain Sciences (CIMeC), University of Trento, Via delle Regole 101, 38123 Trento, Italy; 2 NILab, Bruno Kessler Foundation (FBK), Via delle Regole 101, 38123 Trento, Italy; 3 Department of Psychology, University of California, San Diego, McGill Hall 9500 Gilman Dr, La Jolla, CA 92093-0109 USA

**Keywords:** Prefrontal cortex, Saccades, Working memory, Cognitive control, Activation likelihood estimation, Meta-analytic connectivity modeling

## Abstract

The frontal eye field (FEF) and the inferior frontal junction (IFJ) are prefrontal structures involved in mediating multiple aspects of goal-driven behavior. Despite being recognized as prominent nodes of the networks underlying spatial attention and oculomotor control, and working memory and cognitive control, respectively, the limited quantitative evidence on their precise localization has considerably impeded the detailed understanding of their structure and connectivity. In this study, we performed an activation likelihood estimation (ALE) fMRI meta-analysis by selecting studies that employed standard paradigms to accurately infer the localization of these regions in stereotaxic space. For the FEF, we found the highest spatial convergence of activations for prosaccade and antisaccade paradigms at the junction of the precentral sulcus and superior frontal sulcus. For the IFJ, we found consistent activations across oddball/attention, working memory, task-switching and Stroop paradigms at the junction of the inferior precentral sulcus and inferior frontal sulcus. We related these clusters to previous meta-analyses, sulcal/gyral neuroanatomy, and a comprehensive brain parcellation, highlighting important differences compared to their results and taxonomy. Finally, we leveraged the ALE peak coordinates as seeds to perform a meta-analytic connectivity modeling (MACM) analysis, which revealed systematic coactivation patterns spanning the frontal, parietal, and temporal cortices. We decoded the behavioral domains associated with these coactivations, suggesting that these may allow FEF and IFJ to support their specialized roles in flexible behavior. Our study provides the meta-analytic groundwork for investigating the relationship between functional specialization and connectivity of two crucial control structures of the prefrontal cortex.

**Supplementary Information:**

The online version contains supplementary material available at 10.1007/s00429-023-02641-y.

## Introduction

Owing to the capabilities that likely derive from the massive expansion in the cortical surface area allowed by the folding patterns of the cortex (Van Essen 2007; Zilles et al. 2013), which particularly involved the prefrontal and association cortices (Donahue et al. 2018; Toro et al. 2008), humans possess one of the most complex behavioral repertoires in nature (Mesulam 1998; Miller and Cohen 2001). A fundamental aspect of functional specialization in the human brain is its relationship with cortical neuroanatomy (Van Essen 2007). Microstructural features pertaining to cortical architecture (i.e., cyto- and myelo-architecture), such as cell types and layer organization, are a major determinant of the functional organization of the brain, and they provide important information about regional segregation (Amunts et al. 2020). Over the past 30 years, magnetic resonance imaging (MRI; and in particular, fMRI) became the dominant technique for investigating this organization non-invasively and in vivo (Eickhoff et al. 2018). Although regional delineations inferred based on architectonic criteria (e.g., cytoarchitecture) generally agree well with information gathered from MRI (Amunts and Zilles 2015), such correspondences should be always interpreted with caution. These criteria may be weak predictors of functional organization in highly heterogeneous regions, for example when regions sit at the boundary of different Brodmann areas (BA; Amunts and Zilles 2015; Brodmann 1909). Moreover, this relationship may be affected by strong inter-individual differences, which were not taken into account in most of the previous invasive studies characterized by relatively small sample sizes (Amunts and Zilles 2015). In addition to the previous prevalent invasive and lesion-based approaches, another way of conceptualizing functional organization and, more in general, the relationship between cognitive processes and their neural substrate, emerged from fMRI research with the functional localization approach (Kanwisher 2010). Specialized processes are performed by brain regions that can be reliably identified across individuals with fMRI using standard tasks (hence referred to as functional localizers; Kanwisher 2010; Rosenke et al. 2021). In combination with the functional localization approach, research on structural MRI has shown that, despite the remarkable inter-individual variability in the organization of the gyri and sulci (Desikan et al. 2006; Destrieux et al. 2010; Ono et al. 1990; Petrides 2018), these functional modules can also be localized based on anatomical landmarks (Fischl et al. 2008), which suggests a developmental link between the functional differentiation of brain regions and the mechanisms of cortical maturation (Zilles et al. 2013).

In sum, in the human brain, functional specialization appears to be tightly linked and possibly follows from brain structure, although it remains to be established exactly to what degree this principle holds within specific systems. In the prefrontal cortex (PFC), two lateral structures, the frontal eye field (FEF) and the inferior frontal junction (IFJ) have largely overlapping but complementary roles, being involved in several orchestrating functions such as attention, working memory, cognitive control, and other top-down processes (Baldauf and Desimone 2014; Bedini and Baldauf 2021). In our previous review, we argued that despite these overlaps, it is their patterns of neural selectivity to spatial (FEF) vs non-spatial information (IFJ) that allow the dissociation of their role across these different functions (Bedini and Baldauf 2021). This functional specialization may in turn provide an effective way to localize these regions with MRI. While the FEF has been studied extensively in humans and non-human primates, its precise localization and relationship to sulcal morphology in humans, and correspondence to the macaque FEF has proven to be difficult to establish (Amiez and Petrides 2009; Petit and Pouget 2019; Schall et al. 2020; Tehovnik et al. 2000; but see Koyama et al. 2004). Neuroimaging evidence also suggests that FEF localization may be affected by substantial individual differences (Amiez et al. 2006; Kastner et al. 2007; Paus 1996; see Bedini and Baldauf 2021 for a discussion). The prevailing view is that the human FEF lies in the ventral bank of the superior precentral sulcus (sPCS), near its junction with the superior frontal sulcus (SFS; see Paus 1996 and Vernet et al. 2014, for a meta-analysis and a review of FEF localization, respectively). However, some authors have argued that instead, a region localized ventrally in the dorsal branch of the inferior PCS (iPCS), termed the inferior FEF (iFEF, or sometimes the lateral FEF) may be the putative homolog of the macaque FEF (Kastner et al. 2007; Schall et al. 2020). Moreover, it has been raised the related question of whether the inferior FEF has been under-reported in the fMRI literature (Derrfuss et al. 2012). In topographic mapping studies, peaks corresponding to the iFEF have been already reported (Kastner et al. 2007; Mackey et al. 2017) albeit they were not as consistent as FEF peaks in their presence across subjects and relative localization. Moreover, one study reported activations in the iFEF using a saccadic localizer task, which were clearly segregated from those elicited by a Stroop task (Derrfuss et al. 2012). These analyses were performed in native space on an individual-subject basis, which is a very powerful approach that allows for carefully studying dissociations in adjacent neuroanatomical regions (Fedorenko 2021). The IFJ, a region found ventrally and anteriorly relative to the iFEF, is typically localized near the junction of the iPCS with the inferior frontal sulcus (IFS), sometimes encroaching into its caudal bank (Derrfuss et al. 2005). The IFJ was only much more recently characterized as a separate brain region (based on structural; Amunts and Von Cramon 2006; and functional criteria; Brass et al. 2005) that performs both specialized (Baldauf and Desimone 2014; Bedini and Baldauf 2021; De Vries et al. 2021) and general domain processes (Assem et al. 2020; Derrfuss et al. 2005), in line with the multiple-demand hypothesis (Duncan 2010). Currently, however, due to the interspersed and close arrangement of specialized and multiple-demand regions near the IFJ, common activation foci resulting from various cognitive processes have not been reported yet across experiments (see, however, Assem et al. 2020).

Clearly, there is a need to better characterize the relationship between anatomy and functional specialization within the PFC. This is particularly critical, as the large inter-individual variability in the organization of the prefrontal areas and sulci (Germann et al. 2005; Juch et al. 2005) complicates the interpretation of the results of previous studies and may partly explain the discrepancies in the findings reported across research groups and methods. That such a link can in principle be successfully accomplished has been demonstrated in the visual system, where studies have shown that despite the inter-individual variability in the surface area of the early visual cortex (Benson et al. 2022), specific anatomical landmarks (i.e., sulci) coincide very well with the borders of early visual areas as derived from various sources of data, including cytoarchitecture, retinotopic mapping, myelin content and resting-state fMRI functional connectivity (Abdollahi et al. 2014; Fischl et al. 2008; Glasser et al. 2016; Sereno et al. 1995). Hinds et al. (2008) used surface-based registration methods (Fischl et al. 1999) to identify V1 in new subjects from cortical folding information (i.e., the stria of Gennari) and showed that these methods outperformed volumetric methods in labeling this structure. Similarly, Benson et al. (2012) used folding information to predict visual responses within the striate cortex to a retinotopic mapping fMRI protocol. When moving up into the cortical visual hierarchy, however, the relationships between cortical folding and other neuroanatomical information become more difficult to establish and interpret (Coalson et al. 2018; Glasser et al. 2016). Wang et al. (2015) created a probabilistic atlas of 25 topographic visual areas and showed that anatomical variability (as measured by the variance in gyral-sulcal convexity across subjects) and the overlap of functional activations (measured as peak probability values) were negatively correlated, particularly in higher-order visual areas, suggesting that the former may play an important role in shaping their functional organization. In one of the most comprehensive efforts to parcel the cortical surface with high-resolution non-invasive methods, Glasser et al. (2016) found that the lateral PFC is one of the brain districts where the intrinsic neuroanatomical variability is higher than in the rest of the brain (see also Juch et al. 2005), as measured by a decrease in the test–retest reliability of their multimodal parcellation (MMP1). While the former limitations (i.e., the weaker association between cortical folding and function, and inter-individual variability, which particularly affects volumetric group-level analyses; Coalson et al. 2018) have posed significant challenges to the interpretation of the relationship between cortical folding and functional specialization in higher-order visual regions, several studies have shown that adopting an individual-level approach in defining sulci may bear important implications for understanding cognitive function within the PFC (Amiez et al. 2006; Amiez and Petrides 2018; Derrfuss et al. 2009; Fedorenko 2021; Miller et al. 2021). For example, the study by Frost and Goebel (2012) showed that, by leveraging the former approach and improving the alignment in the cortical folding patterns using a technique termed curvature-driven cortex-based alignment, the overlap in FEF localization increased by 66.7% in the left hemisphere and 106.5% in the right hemisphere compared to volume-based registration in a sample of 10 subjects. These results suggest that the FEF is indeed strongly bound to a macro-anatomical location (Paus 1996), and more generally the presence of a strong structure-to-function relationship in this region (see also Wang et al. 2015). In the study by Derrfuss et al. (2009), which again was carried out using an individual-level approach, 13 out of 14 subjects showed activations localized between the caudal bank of the IFS and the iPCS that corresponded to the anatomical description of the IFJ in a task-switching paradigm. Taken together, these studies point to the need to better characterize the relationship between sulcal morphology and functional specialization within the PFC. This research line may in the future allow predicting functional activity from neuroanatomical information alone, thus accomplishing one of the fundamental goals of contemporary cognitive neuroscience in terms of inferring structure-to-function relationships (Amiez et al. 2006; Osher et al. 2016; Passingham et al. 2002; Saygin et al. 2012). In summary, the organization of the regions localized along the banks of the major sulci of the posterior-lateral PFC, namely the SFS, the sPCS, the iPCS, and the IFS, has yet to be clarified spatially.

In this study, we aimed to accurately localize the FEF and IFJ based on standard fMRI localizer tasks. In particular, we wanted to reassess the precise localization of the FEF in standard space, and its relationship with the localization of the iFEF as inferred using saccadic functional localizers in the light of recent fMRI evidence (see Grosbras et al. 2005, Jamadar et al. 2013, and Paus 1996, for previous meta-analyses using fMRI and PET experiments). Further, we also wanted to re-examine the precise localization of the IFJ in standard space by inferring the convergence of activations across paradigms (Derrfuss et al. 2005; Muhle-Karbe et al. 2016). This information may provide important clues for better-interpreting activations in the posterior-lateral PFC based on combined structural and functional criteria. Coordinate-based meta-analyses offer a convenient way to summarize and model the uncertainty in the activations found across several PET/fMRI experiments (Fox et al. 2014) based on specific paradigms and contrasts of interest, overcoming inter-individual variability, and allow to establish adequately powered brain-behavior relationship. Here, we employed the activation likelihood estimation (ALE) meta-analytic technique to accurately infer the localization of the FEF and IFJ activation peaks in standard space By using the inferred ALE peak coordinates as seeds, we also performed a meta-analytic connectivity modeling (MACM) analysis (Langner and Camilleri 2021) to investigate the coactivation profiles of FEF and IFJ in fMRI studies across paradigms in a data-driven fashion. Overall, the goal of our study is to offer some consensus and anatomical priors to localize these regions with fMRI, and to guide future non-invasive brain stimulation studies. In addition, our study also aims to provide meta-analytic groundwork to investigate the relationship between the functional specialization and connectivity of the FEF and IFJ in large multimodal neuroimaging datasets (e.g., the Human Connectome Project; Van Essen et al. 2013).

## Materials and methods

### Activation likelihood estimation fMRI meta-analysis method

The ALE is a powerful meta-analytic technique that allows for assessing the spatial convergence of the activations reported in the neuroimaging literature (Eickhoff et al. 2012). As a coordinate-based technique, ALE takes as input the activation peaks reported by several independent neuroimaging studies and tests their significance against a null distribution of the foci across the whole brain (Eickhoff et al. 2012). This ALE feature is particularly useful given that in the neuroimaging literature results are usually reported and summarized as *x*, *y*, *z* coordinates in standard space (Talairach or MNI), rather than as full activation maps accompanied by a statistical summary of the effect sizes, and even more rarely shared in that form (for important initiatives in neuroimaging data sharing see, however, NeuroVault: https://neurovault.org/, Gorgolewski et al. 2015, and OpenNeuro: https://openneuro.org/, Markiewicz et al. 2021, among other initiatives). This aspect becomes crucial in the case of brain regions that may be under-reported in the fMRI literature (such as the iFEF; Derrfuss et al. 2012; Kastner et al. 2007) or which only recently began to be included in the brain atlases taxonomy (such as the IFJ; Bedini and Baldauf 2021; Sundermann and Pfleiderer 2012). Here, we exploited this ALE feature by applying this technique to analyze two independent collections of fMRI studies performed over the last 30 years with the primary aim of accurately inferring FEF and IFJ localization in MNI152 space using the GingerALE software (v. 3.0.2; https://www.brainmap.org/ale/). In the ALE procedure, each set of foci reported in a study is modeled as a three-dimensional Gaussian distribution centered around the coordinates and whose width is determined based on the experiment sample size (Eickhoff et al. 2012). Larger sample sizes result in tighter Gaussians, which reflects lower uncertainty about the ‘true’ location reported, whereas lower samples lead to larger Gaussians that are more spread around the respective peak coordinates, thus conveniently reflecting lower confidence about their corresponding locations. These activations are then combined into a modeled activation map for each experiment of a study. Importantly, in the revised ALE algorithm, within-study effects that could result from the summation of adjacent foci are minimized, so that studies that reported activation in a higher number or more densely organized foci would not drive the ALE results disproportionately (Turkeltaub et al. 2012). By computing the union of all these modeled activation maps, an ALE score for each voxel in the brain is obtained (Eickhoff et al. 2012). The significance of these scores is then assessed by comparing them with the null distribution obtained by randomly reassigning the modeled activations across the whole brain with a permutation approach. Finally, the thresholded p-values are usually corrected for multiple comparisons using either voxel-level or cluster-level family-wise error (FWE). The use of uncorrected p-values and false discovery rate is instead generally not advised since it can lead to spurious findings (Eickhoff et al. 2016).

The present meta-analysis focused on specific cognitive functions (described more in detail in the 'Study selection criteria') in which the FEF and IFJ and the associated brain networks are relatively well-known to be involved. More specifically, in what we will refer to in the following as the ‘FEF sample’, we applied the ALE technique to several independent fMRI studies requiring the planning and execution of visually guided and voluntary eye movements, as a considerable number of previous studies clearly showed that these types of tasks elicit activation in the FEF, and other eye fields (for previous meta-analyses see Cieslik et al. 2016; Grosbras et al. 2005; Jamadar et al. 2013; Paus 1996). In particular, tasks requiring the execution of prosaccades and antisaccades contrasted against a fixation baseline are the most prevalent and consensually established FEF functional localizer in the human fMRI literature (Amiez et al. 2006; Amiez and Petrides 2018; Kastner et al. 2007). In the case of the ‘IFJ sample’, it is arguably more difficult to pinpoint a widely employed functional localizer for this region, We, therefore, anticipate that in this sample we analyzed data from a more heterogeneous collection of fMRI studies investigating covert attention, working memory, and cognitive control across a wider range of paradigms.

### Study selection criteria

The selection criteria of the sample of studies for the present meta-analysis followed the best-practice recommendations and guidelines by Müller et al. (2018). Multiple bibliographic searches were performed between May 2019 and January 2021 (cutoff date). A final search was conducted with the same criteria and cutoff dates (i.e., January 1, 1990–January 1, 2021) by the first author MB to comply with the updated PRISMA guidelines (Page et al. 2021) and as a sanity check. The selection procedure is reported in Figures S1 and S2 (created based on the PRISMA flow diagram; Page et al. 2021), which refer to the ‘FEF sample’ and the ‘IFJ sample’, respectively. All the bibliographical searches were carried out using the Web of Science (https://www.webofscience.com). We searched records in the Web of Science Core Collection using the keywords ‘fMRI’ AND ‘frontal eye field’ (all fields) in the first instance, and ‘fMRI’ AND ‘inferior frontal junction’ (all fields) in the second. We complemented these results with other sources (Google Scholar, personal collection of articles and references cited by the studies retrieved) by one of the authors (MB). In the FEF sample, our search identified a total of 711 records, from which we removed all the review papers. 665 records were further screened, and 470 of these were sought for retrieval to assess their adequacy with respect to the inclusion criteria (described below). In the IFJ sample, 375 results were identified, from which, after removing the review papers, 356 records were further screened, and 142 of these were sought for retrieval to assess their adequacy with respect to our inclusion criteria. The general inclusion criteria consisted of the following. Each study selected: 1. Reported coordinates in standard space (either MNI or Talairach); 2. Was an fMRI study (we decided to not include PET studies as our goal was to keep our samples as homogeneous as possible in terms of the signal measured, the spatial resolution and analysis pipelines (Botvinik-Nezer et al. 2020) to make our results specific to the fMRI field); 3. Performed on a scanner of 3 T or higher field; 4. Tested and reported results from healthy adults (18–60 years old; or an appropriate control group in the case of clinical studies); 5. The study acquired whole-brain fMRI data or with a FOV that was sufficiently large to cover the posterior frontal lobe (see Supplementary Tables 1 and 2 for the FOV of each study). The last group of inclusion criteria is specific to each sample (the FEF or the IFJ) and is primarily related to the type of experimental paradigm utilized in the fMRI study and the specific contrasts analyzed. Here, we strived to find a balance that would adequately represent the various localization methods that have been pursued in the fMRI literature, while also assigning a higher weight in the sample to the more standardized and replicated localization approaches.

### FEF sample inclusion criteria

The human FEF is a well-characterized region in the fMRI literature (Bedini and Baldauf 2021), although some uncertainties persist regarding the correspondence of its localization obtained from fMRI compared with other methods (i.e., brain stimulation; Vernet et al. 2014) and with the macaque FEF (Koyama et al. 2004; Petit and Pouget 2019). The region is crucially involved in the top-down control of eye movements and spatial attention (Astafiev et al. 2003; Beauchamp et al. 2001; Corbetta et al. 1998; de Haan et al. 2008), and it is considered a prominent node of the dorsal attention network (Corbetta and Shulman 2002; Fox et al. 2006; Yeo et al. 2011). A very simple and time-efficient yet effective way to localize the FEF with fMRI is to have participants perform an experimental block of visually guided saccades toward an unpredictable peripheral target and contrast this activation with a fixation block (Amiez et al. 2006). The resulting activations—usually found near the junction of the SFS and the sPCS—are then assumed to correspond to the FEF (Paus 1996). However, depending on the statistical thresholds and analytical approach adopted, in addition to this superior cluster, often this type of contrast reveals a more widespread pattern of activity along the banks of the iPCS (Beauchamp et al. 2001; Kastner et al. 2007; Luna et al. 1998). Therefore, this localization method does not seem to have adequate functional specificity if not combined with the additional anatomical criteria mentioned above. Building on this approach, the antisaccade task and its neural mechanisms have been extensively studied in the non-human primate neurophysiology literature (Munoz and Everling 2004), and this task has been employed as a measure of inhibitory control in healthy and clinical populations in humans (Hutton and Ettinger 2006). Briefly, in the antisaccade task, the subject is required to keep fixation until a visual target appears and to look at its mirror location. Computationally, this requires at least two mechanisms: the first one inhibits a reflexive saccade towards the visual onset, and the second is responsible for executing a saccade towards the opposite location (the endpoint is in this case endogenously generated; Munoz and Everling 2004). fMRI studies comparing the regions involved in prosaccades vs antisaccades have found overlapping activations in the FEF, although the antisaccade task recruits additional regions that seem to reflect the greater executive demands of this task (McDowell et al. 2008). Within FEF, there is also increased activity in the antisaccade compared to the prosaccade task, which is particularly evident during the preparatory phase (Curtis and Connolly 2008; DeSouza et al. 2003: Fernandez-Ruiz et al. 2018; Jarvstad and Gilchrist 2019). Based on these results, it could be hypothesized that contrasting antisaccade vs and prosaccade trials may offer better specificity to localize clusters of activity within the FEF compared to the prosaccade vs fixation blocked design described earlier. Previous studies did not address this hypothesis directly, and it still needs to be tested in a within-subject design by examining the activation topographies at the individual subject level. Finally, in modified versions of the spatial cueing paradigm (e.g., Fan et al. 2005), univariate analyses contrasting valid vs neutral/invalid trials are often used to localize all the main regions belonging to the dorsal attention network, which are subsequently used as ROIs for functional and effective connectivity analyses (for examples see Vossel et al. 2012, and Wen et al. 2012). It can be argued that, even though these adaptations are not generally employed as independent functional localizers for the FEF, they may be well adept at isolating this region under the assumption that covert and overt shifts of spatial attention have a shared and overlapping source in this region, which seems well supported by fMRI (Astafiev et al. 2003; Beauchamp et al. 2001; Corbetta et al. 1998; de Haan et al. 2008; Jerde et al. 2012) and comparative evidence (Buschman and Miller 2009; Moore and Fallah 2001; reviewed in Fiebelkorn and Kastner 2020). Indeed, the studies that directly investigated this question generally reported a strong degree of spatial overlap, although they also suggest that the signal measured in covert paradigms tends to be weaker than in overt tasks (Beauchamp et al. 2001; de Haan et al. 2008) and thus possibly less robust across fMRI data analysis pipelines (Botvinik-Nezer et al. 2020). Thus, an open question is whether oculomotor and covert spatial attention tasks are equally efficient in localizing the FEF.

In summary, for the reasons introduced above, we included in the FEF sample all the studies that investigated the planning and execution of visually guided and voluntary eye movements (prosaccades and antisaccades) as well as covert spatial attention using both blocked and event-related designs (see Figure S1 for an overview of the selection procedure following the PRISMA2020 guidelines; Page et al. 2021), analyzing mainly the following contrasts: 1. prosaccades > fixation; 2. antisaccades > fixation; 3. prosaccades & antisaccades > fixation; 4. antisaccades > prosaccades; 5. valid > neutral/invalid trials. Combining these contrasts allowed us to carry out our main analysis complemented by three control analyses, respectively, designed to replicate a previous study (Cieslik et al. 2016) and to investigate two additional research questions (see Supplementary Information p. 8). In our main localizer analysis, we pooled together all studies that reported at least a contrast related to the planning and execution of prosaccades and antisaccades contrasted with a fixation baseline.

### IFJ sample inclusion criteria

In contrast to the FEF, the IFJ does not have a well-established homolog in the macaque (Bedini and Baldauf 2021; see, however, Bichot et al. 2015, 2019, and Neubert et al. 2014) and its role started to be investigated only much more recently with fMRI (Brass et al. 2005). Its functional profile remains to date not well understood and is characterized by a remarkable functional heterogeneity (Muhle-Karbe et al. 2016; Ngo et al. 2019). Consistent with this idea, recent high-resolution fMRI studies showed that the IFJ (and in particular, the posterior IFJ as defined according to the MMP1 by Glasser et al. 2016) belongs to the core multiple-demand system of the brain (Assem et al. 2020), which identifies a set of regions that are engaged in multiple processes often across different cognitive domains (Duncan 2010). This particular position in the cognitive processing architecture arguably poses a severe challenge in trying to define a gold standard for an fMRI localization method for this region, which would allow for effectively segregating it from adjacent coactive regions. Several promising approaches to localize the IFJ at the individual level have nevertheless previously been reported from different research groups ranging from attention and working memory to cognitive control paradigms (Baldauf and Desimone 2014; Derrfuss et al. 2012; Zanto et al. 2010). The studies led by Brass, Derrfuss and colleagues were critical in establishing the IFJ as a region involved in task preparation and more generally in cognitive control (reviewed in Brass et al. 2005). In more recent studies, these processes are reflected in the distinct components of executive functions, such as updating and shifting the task set, which consistently recruit this region (Nee et al. 2013; Rodríguez-Nieto et al. 2022; Worringer et al. 2019).

Based on the evidence introduced above, in the IFJ sample, we decided to include attentional (i.e., rapid serial visual presentation (RSVP) and endogenous cueing paradigms), working memory (primarily n-back paradigms; Rottschy et al. 2012), and cognitive control paradigms (i.e., task-switching and Stroop tasks; Worringer et al. 2019; see Figure S2 for an overview of the selection procedure following the PRISMA2020 guidelines; Page et al. 2021). These inclusion criteria were based on Derrfuss et al. (2005), who investigated switching and Stroop paradigms, and we extended them to attentional and working memory paradigms that also tap cognitive control. The main contrasts analyzed were, therefore, quite heterogeneous, but can be broadly grouped into the following primary ones: 1. Oddball > Target trials in covert attention paradigms (e.g., RSVP paradigms); 2. Functional connectivity with a seed perceptual region (e.g., V4, V5, FFA) in the Attend > Ignore condition in n-back paradigms; 3. Switching > Repetition trials in task-switching paradigms; 4. Incongruent > Congruent trials in Stroop paradigms. We carried out exploratory ALE contrast analyses to examine potential spatial discrepancies between the IFJ peaks derived from splitting up the localizer sample according to the main cognitive function investigated (see Supplementary Information p. 9 and 20–22).

In conclusion, the final sample of the included papers for our ALE meta-analysis was *n* = 51 for the FEF, and *n* = 30 for the IFJ sample (see Tables S1 and S2 in the Supplementary Information for a summary of the studies, respectively). The number of experiments was 35 for the FEF localizer sample, and 32 for the IFJ localizer sample. Both sample sizes were within the recommended range (i.e., a minimum of 20 experiments) to have adequate statistical power with ALE as derived from empirical simulations (Eickhoff et al. 2016).

### Activation likelihood estimation procedure

All the foci from the experiments included were mapped from Talairach to the MNI152 space using the function provided by GingerALE (v. 3.0.2; Eickhoff et al. 2012; Lancaster et al. 2007; see Supplementary Information p. 8 for details about this step).

For the main localizer analyses (FEF and IFJ localizer samples), the ALE parameters were set to 5000 threshold permutations and a voxel-level FWE of 0.01 was applied (Eickhoff et al. 2016) with a minimum cluster size of 50 mm^3^ (corresponding to 6 voxels). Compared to cluster-level FWE inference, which can only allow inferring that a given cluster is above a significance threshold as a whole, but critically, not that any putative region that is included in the cluster is individually significant on its own, voxel-level FWE allows to more readily interpret all the cluster extent as well as its peak location from the main localizer samples anatomically (Eickhoff et al. 2016). Moreover, our sample sizes ensured that these clusters would not be driven by a contribution exceeding 50% of any individual study (Eickhoff et al. 2016), therefore allowing us to interpret individual voxel ALE values as a proxy for the most active location across experiments. When retrieving the relevant foci, we first grouped the studies by a subject group rather than by experiment. This was done because grouping by subject group further minimizes within-study effects (Turkeltaub et al. 2012). When a single experiment reported multiple contrasts of interest, we, therefore, pooled them under the same subject group. We note that however, in all cases in which the studies reported more than one contrast of interest they were drawn from the same experiment (with very few exceptions; see Tables S1 and S2), so our strategy did not unfairly pool together partially independent observations and was practically almost equivalent to grouping by experiment. When an experiment failed to report significant activation for some ROIs, we used the lower number of subjects that had above threshold activations in all ROIs from a contrast of interest if this information was available. To validate the results of the main ALE analyses and to further assess the reliability of the ALE peaks found, we also carried out a leave-one-experiment-out procedure (LOEO; Eickhoff et al. 2016) on the main FEF and IFJ localizer samples using the same foci grouping strategy. Since we found identical ALE peaks as in the main ALE analyses using 1000 threshold permutations, we performed the LOEO procedure with the same parameter to reduce computational times.

### Activation likelihood estimation control analyses

We carried out two control analyses in the FEF and IFJ localizer samples to rule out potential biases in our main ALE results. As we were mainly interested in inferring the localization of the FEF and IFJ, we decided to include studies with partial brain coverage. We motivated this choice by accepting the tradeoff between having access to a larger sample of experiments for these regions, as opposed to having less sensitivity in detecting other regions that are consistently active during the tasks included (which, however, are not the main focus of the present study), but not reported simply due to the lack of whole-brain brain coverage. The inclusion of studies with partial brain coverage is, however, generally not recommended in ALE analyses (Müller et al. 2018). In our first set of control analyses, we have, therefore, excluded those studies based on the FOV parameters reported in the study (see Tables S1 and S2) or the author’s description of the fMRI data acquisition. This led to the exclusion of two experiments from the FEF sample (Amiez and Petrides 2018, and Berman et al. 1999), and one study in the IFJ sample (Sreenivasan et al. 2014). For this control analysis, the ALE parameters were set to 5000 threshold permutations and a voxel-level FWE of 0.01 with a minimum cluster size of 50mm^3^. The second set of control analyses was carried out to rule out another potential source of biases, namely ROI analyses (Müller et al. 2018). These analyses imply restricting the assessment of statistical significance by some form of spatial masking. In this case, we excluded all the studies that either carried out ROI analyses or that only reported their results for specific ROIs (or even only FEF and IFJ foci; see Tables S1 and S2). This led to an important decrease in both the FEF and IFJ localizer sample sizes (to 16 and 22 eligible experiments, respectively). The ALE parameters were set to 5000 threshold permutations and a voxel-level FWE of 0.01 with a minimum cluster size of 50 mm^3^, as in our main localizer analyses.

### Comparison method of the ALE clusters and peaks with previous coordinate-based meta-analyses, relationship to macro-anatomical information and the MMP1

To interpret our results more carefully, we compared the clusters obtained from our ALE main localizer analyses with the results from previous meta-analyses results and brain atlases (Derrfuss et al. 2005; Glasser et al. 2016; Klein and Tourville 2012; Paus 1996). First, we described the anatomical location of each cluster and assigned the corresponding BA using the Talairach Daemon in GingerALE (Lancaster et al. 2000). Second, to compare our results with previous meta-analyses (Derrfuss et al. 2005; Paus 1996), we mapped our ALE peaks to the Talairach space using the transformation developed by Lancaster et al. (2007) with GingerALE (MNI (FSL) to Talairach; see Table 3). Third, to relate our results to surface-based atlases (Klein and Tourville 2012; Glasser et al. 2016), we followed two distinct approaches. The Mindboggle 101 atlas (Klein and Tourville 2012) describes the macro-anatomical organization of the human brain as delineated by sulcal and gyral information. The atlas was recently mapped to the MNI152 non-linear symmetric template (Manera et al. 2020), thus we manually imported this atlas in FSL and in FSLeyes as described in this GitHub repository: The-Mindboggle-101-atlas-in-FSL. We assigned one of the Mindboggle 101 labels to each of the ALE peak coordinates using the atlasquery command-line tool with FSL (v. 6.0.3; Jenkinson et al. 2012). For atlases that were released and best interpreted in a surface format, such as the MMP1 (Glasser et al. 2016; see Coalson et al. 2018 for an in-depth discussion), we instead employed the mapping technique developed in Wu et al. (2018) to register our ALE results from the MNI152 space to FSaverage (Fischl et al. 1999). A version of the MMP1 mapped to the FSaverage surface was made available using the method described in Mills (2016; https://figshare.com/articles/dataset/HCP-MMP1_0_projected_on_fsaverage/3498446). Once we mapped the ALE clusters to this surface, we also mapped the MNI152 coordinates corresponding to each ALE peak to a vertex on the inflated surface and we assigned each of these to the respective MMP1 labels (Table 3). To describe the anatomical labels associated with each ALE cluster using more specific labels (compared to the Talairach Daemon), we used a volumetric version of this atlas for convenience. The source files that were used to import the atlas are the same as in Huang et al. (2022). The volumetric version of the MMP1 was manually imported in FSLeyes as described in this GitHub repository: The-HCP-MMP1.0-atlas-in-FSL.

### Meta-analytic connectivity modeling method

We exploited the ALE peaks obtained from the main localizer analyses to perform a data-driven analysis of the coactivation patterns of the FEF and the IFJ across the whole brain to uncover their task-based fMRI functional connectivity fingerprint (Langner and Camilleri 2021). We, therefore, retrieved all the papers matching specific criteria (described below) from the BrainMap database using Sleuth (https://www.brainmap.org/sleuth/; Fox and Lancaster 2002), and we analyzed these foci by employing the MACM technique (Langner and Camilleri 2021). This technique leverages the ALE algorithm and allows inferring all the regions that coactivate with a given seed region that is selected a priori. This analysis also allowed us to perform a reverse inference on these coactivation patterns (Poldrack 2011). More specifically, we sought to functionally decode and characterize the various behavioral domains that are significantly associated with each of these using a standardized taxonomy (Fox et al. 2005) via the Mango software (v. 4.1) behavioral analysis plugin (v. 3.1; Lancaster et al. 2012).

The studies were retrieved from the BrainMap database using Sleuth according to the following fields (all linked using the ‘AND’ operator): in the Experiment field, the “context” field was set to “normal mapping”, in the “activation” field we searched for “activations only”, with “Imaging modality” being set to “fMRI”. Finally, four separate searches were conducted in the BrainMap database by setting the “locations” field as corresponding to each left hemisphere (LH) and right hemisphere (RH) seed region (LH FEF, RH FEF, LH IFJ, and RH IFJ). We first transformed each seed location from MNI152 to Talairach space (which is the standard in Sleuth and also used internally by Mango’s behavioral plugin) using the transformation by Lancaster et al. (2007; the FSL transformation) and we created cuboid seeds of 6 mm centered around the respective ALE peaks. With these criteria, we were able to retrieve a range of 19 to 53 studies across seed locations. We retrieved 26 studies (27 experiments) for the LH FEF seed, 19 studies (19 experiments) for the RH FEF seed, 53 studies (59 experiments) for the LH IFJ, and 31 studies (31 experiments) for the RH IFJ. We note that the different number of studies retrieved possibly reflects a combination of the increased base probability of finding activations within a specific ROI (Langner et al. 2014) but also the fact that some ROIs tend to participate in multiple functional networks (Langner and Camilleri 2021), which seems to fit well with the role of the IFJ in the frontoparietal network (Cole et al. 2013). Crucially, these sample sizes allowed for adequately powered inference using ALE (Eickhoff et al. 2016). Therefore, we used all the foci retrieved separately from each seed location as inputs for GingerALE. The ALE parameters were set to an 0.001 uncorrected *p* value, 1000 threshold permutations, and a cluster-level FWE of *p* < 0.01. In the functional decoding analysis, we used the same 6 mm cuboid seeds as in the MACM analysis centered around the respective FEF and IFJ ALE peaks. Associations with behavioral domains were considered statistically significant when their *z* score was ≥ 3, corresponding to a threshold of *p* < 0.05 (Bonferroni corrected).

## Results

### FEF and IFJ localizer samples ALE main clusters

**Fig. 1:**
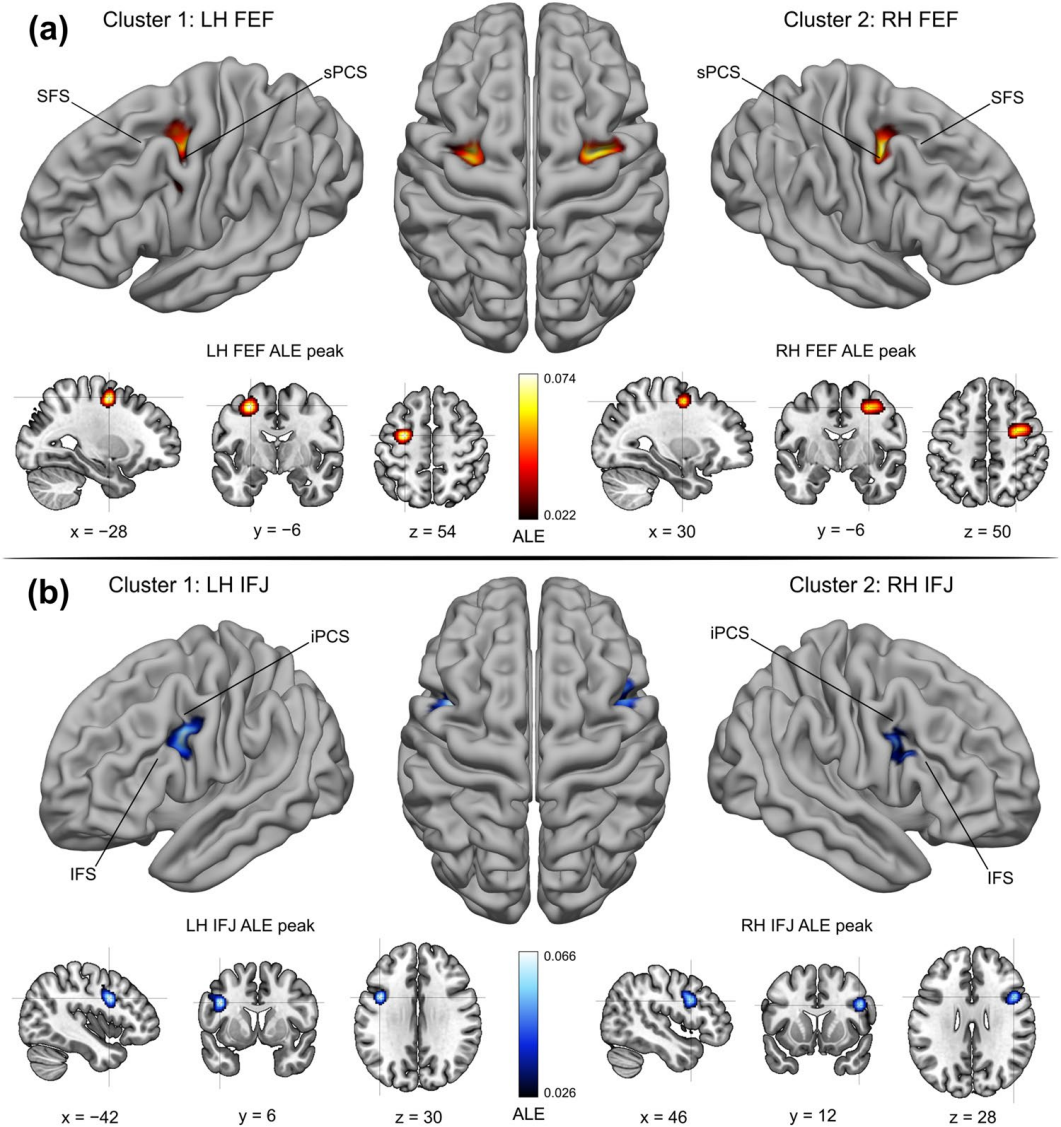
FEF and IFJ localizer samples—ALE main clusters. **A** ALE results from the FEF localizer sample. Two main clusters were found in the posterior dorsolateral PFC, which corresponds to the description of the anatomical location of the FEF (Paus 1996; Vernet et al. 2014). The FEF peaks were localized at the junction of the sPCS and the SFS, in the anterior (in the LH) and posterior (in the RH) banks of the sPCS. **B** ALE results from the IFJ localizer sample. Two main clusters were found in the posterior ventrolateral PFC, and their respective peaks were localized along the posterior bank of the iPCS, near its ventral junction with the IFS. The location of these peaks and the corresponding MNI152 coordinates match the description of the IFJ (Derrfuss et al. 2005; Muhle-Karbe et al. 2016)

In the FEF localizer sample, the activations converged most strongly in two main clusters localized in the left and right posterior dorsolateral PFC. Two ALE peaks were found near the junction of the sPCS with the SFS, localized in the anterior (in the LH) and posterior (in the RH) banks of the sPCS (Fig. 1). These peaks match well the classical description of the human FEF as inferred with fMRI (Petit and Pouget 2019; Vernet et al. 2014). Our LOEO procedure overall confirms the reliability of the localization of these ALE peaks (see Table 3; LH: 26/35; RH: 23/35). In the IFJ localizer sample, the activations converged most strongly in two main clusters localized in the left and right posterior ventrolateral PFC. These clusters extended both in the dorsal and ventral portion of the iPCS, partially encroaching on the IFS (see Fig. 1). The cluster in the right hemisphere was slightly smaller and spatially more focused compared to the cluster in the left hemisphere. Crucially, in both clusters, we found that the ALE peaks were localized along the posterior bank of the iPCS, near its ventral junction with the IFS, which closely matches the description of the IFJ (Derrfuss et al. 2005; Muhle-Karbe et al. 2016). Again, our LOEO procedure overall suggests that these ALE peaks are highly reliable across experiments (see Table 3; LH: 31/32; RH: 24/32).

### FEF localizer sample ALE results—FEF lateral peak and other significant clusters

**Fig. 2:**
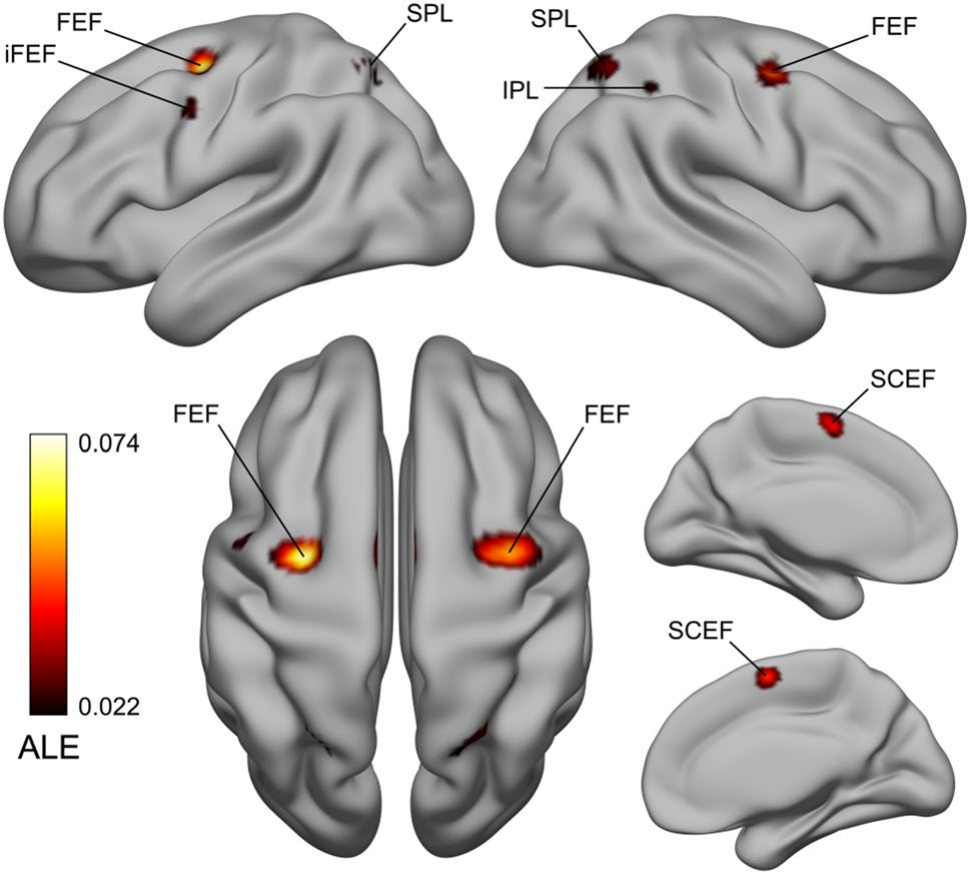
FEF localizer sample ALE results—FEF lateral peak and other significant clusters. In the FEF localizer analysis, we also found a lateral peak in the LH, which was localized near the bank of the iPCS, dorsal to its junction with the IFS, corresponding to the iFEF (Derrfuss et al. 2012; Kastner et al. 2007). It is unclear whether this region should be considered part of the FEF proper (Glasser et al. 2016; Mackey et al. 2017). We found three other significant clusters localized in the supplementary and cingulate eye field (SCEF) and the dorsal cingulate motor cortex, and the precuneus/superior parietal lobule (SPL) and the right anterior intraparietal area. These areas form some of the core control structures of the oculomotor network (Coiner et al. 2019)

In the left hemisphere, we also found a lateral peak within the main FEF cluster, which was localized on the bank of the iPCS, dorsal to its junction with the IFS (see Fig. 2). This lateral peak corresponds to what has been previously referred to as the inferior or the lateral FEF (Derrfuss et al. 2012; Kastner et al. 2007; Luna et al. 1998). In addition to the main FEF clusters in the left/right PFC, the ALE technique revealed three other consistently activated clusters. These clusters were localized in the medial frontal gyrus and the left/right posterior parietal cortex (Table 1). The cluster in the medial frontal gyrus comprised the SCEF (Amiez and Petrides 2009) and the dorsal cingulate motor cortex (Glasser et al. 2016). In the posterior parietal cortex, two bilateral superior clusters spanned the precuneus and the SPL (Scheperjans et al. 2008a, b), and an additional cluster was found in the right anterior intraparietal area (Glasser et al. 2016; Numssen et al. 2021).

**Table 1:** FEF localizer sample ALE results

Cluster	Macroanatomical location	Hemi	MNI152 coordinates			ALE value	Volume (mm³)	BA
			*x*	*y*	*z*			
1	Precentral Gyrus	L	−28	−6	54	0.0736	4960	6
	Precentral Gyrus	L	−48	−2	40	0.0293		6
2	Precentral Gyrus	R	30	−6	50	0.0624	4304	6
3	Medial Frontal Gyrus	L/R	0	0	58	0.0548	3088	6
4	Precuneus	L	−22	−58	56	0.0418	1568	7
5	Precuneus	R	24	−60	56	0.0376	1104	7
6	Inferior Parietal Lobule	R	36	−46	48	0.0262	176	40

### IFJ localizer sample ALE results—other significant clusters

**Fig. 3:**
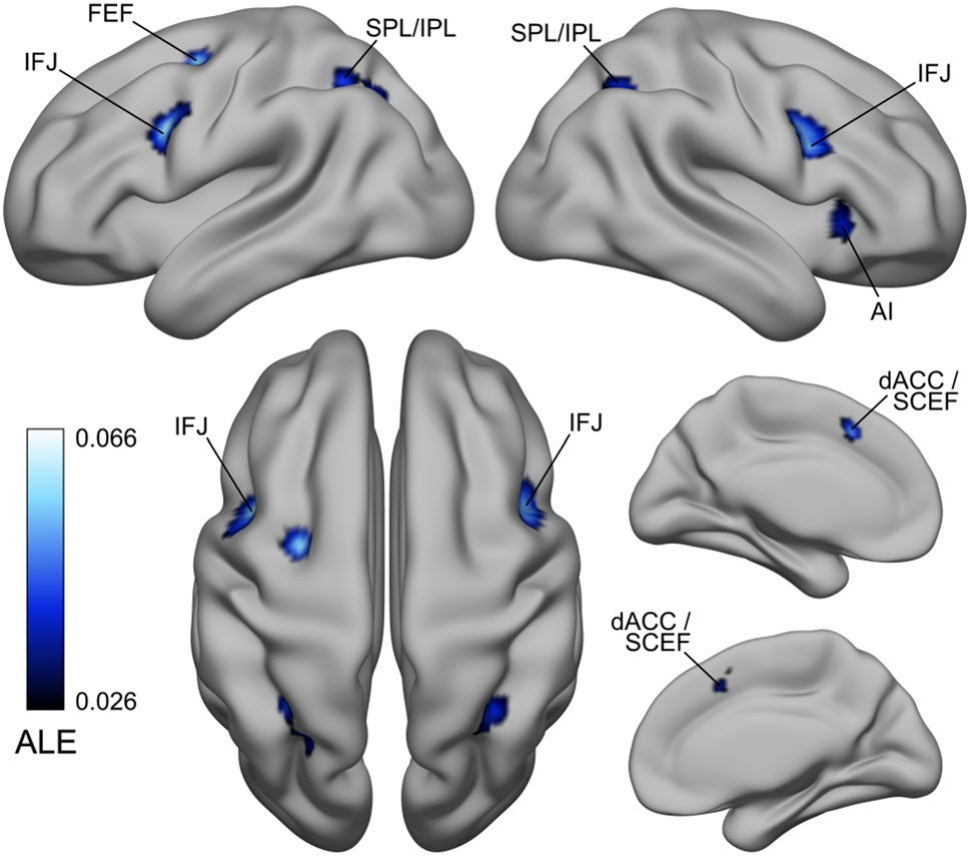
IFJ localizer sample ALE results—Other significant clusters. In addition to the bilateral IFJ clusters, we found significant activations in the dACC/SCEF, the left FEF, in two clusters in the insular cortex and claustrum (not visible in the LH), and finally, in the SPL/IPL. Given that these areas were activated across different paradigms, we suggest that they could be associated with the “encoding and updating of task-relevant representations” as first hypothesized by Derrfuss et al. (2005; see also Rodríguez-Nieto et al. 2022). These areas mostly belong to the frontoparietal network (Yeo et al. 2011)

In addition to the main IFJ clusters in the left/right PFC, we found seven consistently activated clusters forming a broad frontoparietal network (see Fig. 3). In the frontal cortex, the first cluster was localized in the dorsal anterior cingulate cortex (dACC) and SCEF (Amiez and Petrides 2009; Glasser et al. 2016), a second in the left precentral gyrus (within the putative FEF), and finally, two other clusters were localized in the bilateral insular cortex and claustrum (Table 2). Posteriorly, we also found a cluster in the left SPL/inferior parietal lobule (IPL), and a smaller cluster in the right SPL/IPL (Numssen et al. 2021; Scheperjans et al. 2008a, b).

**Table 2:** IFJ localizer sample ALE results

Cluster	Macroanatomical location	Hemi	MNI152 coordinates			ALE value	Volume (mm³)	BA
			*x*	*y*	*z*			
1	Precentral Gyrus	L	−42	6	30	0.0658	3488	6
2	Inferior Frontal Gyrus	R	46	12	28	0.0597	3016	9
3	Medial Frontal Gyrus	L	−2	18	44	0.0574	2328	6
4	Superior Parietal Lobule	L	−30	−54	48	0.0445	1504	7
	Precuneus	L	−26	−66	44	0.0388		7
5	Precentral Gyrus	L	−28	−4	54	0.0578	1208	6
6	Superior Parietal Lobule	R	34	−56	48	0.0408	800	7
7	Claustrum	R	32	22	−2	0.0386	728	NA
8	Claustrum	L	−30	18	2	0.0343	424	NA

NA = Not Available

### Control analyses ALE results

The results of the control analysis excluding studies with partial brain coverage replicated our main localizer results, both in terms of the inferred ALE peaks and of the number and anatomical localization of the significant clusters. In the FEF sample, the ALE peaks coincided with the main analysis, and the only difference that emerged was a minor shift of the left iFEF peak (from MNI152: − 48, − 2, 40 to − 46, − 2, 38). In the IFJ sample, the ALE peaks again coincided with the results of our main analysis. The results of the second control analysis where we excluded all ROI analyses again matched well with our main results in terms of the inferred ALE peaks but had important differences regarding the number and anatomical localization of the significant clusters. The results of this control analysis are reported in the Supplementary Information (see Tables S6 and S7 for the FEF and IFJ samples, respectively). In the FEF sample, the cluster with the highest ALE value was now localized primarily in the left medial frontal gyrus, including the SCEF. The second and third larger clusters were, however, again localized in the left and right FEF, respectively, and were of the same size approximately. The inferred FEF ALE peaks were almost identical to our main results (LH main: − 28, − 6, 54 vs no ROI control: − 28, − 6, 56; and RH main: 30, − 6, 50 vs no ROI control: 28, − 6, 50; all the coordinates are in MNI152 space). However, in contrast to our main analysis results, the left FEF cluster did not spread onto the iPCS and there was not any peak corresponding to the iFEF. Interestingly, we also found a second peak in the right FEF cluster, which was lateral relative to the first, although still localized within the sPCS (40, − 4, 50). In the IFJ sample, the most prominent clusters were localized in the left and right IFJ, replicating our main analysis results. Their ALE peak exactly matched those from our main results in the left hemisphere (− 42, 6, 30) but had some slight differences in the right hemispheres, where we found two peaks with similar ALE values (main: 46, 12, 28 vs no ROI control: 46, 10, 26, and 42, 8, 30). This indicates some residual variability in the localization of the right IFJ, which was not detectable in our main analysis results.

### Spatial relationship of the main FEF and IFJ ALE clusters and peaks with previous coordinate-based meta-analyses, macro-anatomical information and the MMP1

The comparison of the FEF and IFJ ALE peaks from the localizer samples analyses overall shows good spatial correspondence with results from previous meta-analyses and the MMP1, but with some important differences that are worth examining in detail (see Table 3). The FEF ALE peaks from our results are localized much more medially and posteriorly relative to the results reported in Paus (1996), highlighting marked spatial differences with this landmark FEF meta-analysis. In contrast, the IFJ ALE peaks are virtually identical to those reported in the study by Derrfuss et al. (2005; with slightly less agreement in the RH), where the authors also employed the ALE technique in one of its earlier implementations. Macro-anatomically, according to the Mindboggle 101 atlas (Klein and Tourville 2012; Manera et al. 2020), the LH FEF and RH FEF peaks lie within the caudal middle frontal gyrus (in BA 6) and not in the precentral gyrus, as previously assumed based on non-human primate evidence (Bruce et al 1985; Schall et al. 2020, and Tehovnik et al. 2000, for reviews). These results are consistent with the few pieces of evidence available on the delineation of this region based on cytoarchitecture in *post-mortem* studies (Rosano et al. 2003; Schmitt et al. 2005). While the left IFJ ALE peak was found in the precentral gyrus (in BA 6), interestingly the right IFJ ALE peak was instead localized within the pars opercularis (in BA 9). The distinctive architecture of the IFJ remains elusive, but these peaks agree with the evidence that this area is localized in several Brodmann areas (BA6, 8, 9, 44 and 45), and may correspond to a specific cyto- and chemo-architecture found dorsal to BA44 (Amunts and Von Cramon 2006; for a recent fine-grained analysis of its architecture see Ruland et al. 2022).

**Table 3:** Comparison of the ALE peaks with previous meta-analyses and brain atlases

Cluster	Hemi	ALE peaks						Previous meta-analyses (Talairach space)							Brain atlases		LOEO results
		MNI152 coordinates			Talairach coordinates			Paus (1996)				Derrfuss et al. (2005)			Mindboggle 101	MMP1	
		*x*	*y*	*z*	*x*	*y*	*z*	*x*	*y*	*z*		*x*	*y*	*z*	Label	Label	ALE peak ratio
FEF	L	−28	−6	54	−27.73	−9.61	51.32	−32	−2	46		//	//	//	Caudal Middle Frontal Gyrus	i6-8	26/35
FEF	R	30	−6	50	27.17	−9.86	48.03	31	−2	47		//	//	//	Caudal Middle Frontal Gyrus	6a	23/35
IFJ	L	−42	6	30	−40.87	3.27	30.37	//	//	//		−40	4	30/32	Precentral Gyrus	6r	31/32
IFJ	R	46	12	28	42.43	8.35	29.4	//	//	//		44	10	34	Pars Opercularis	6r	24/32

**Fig. 4:**
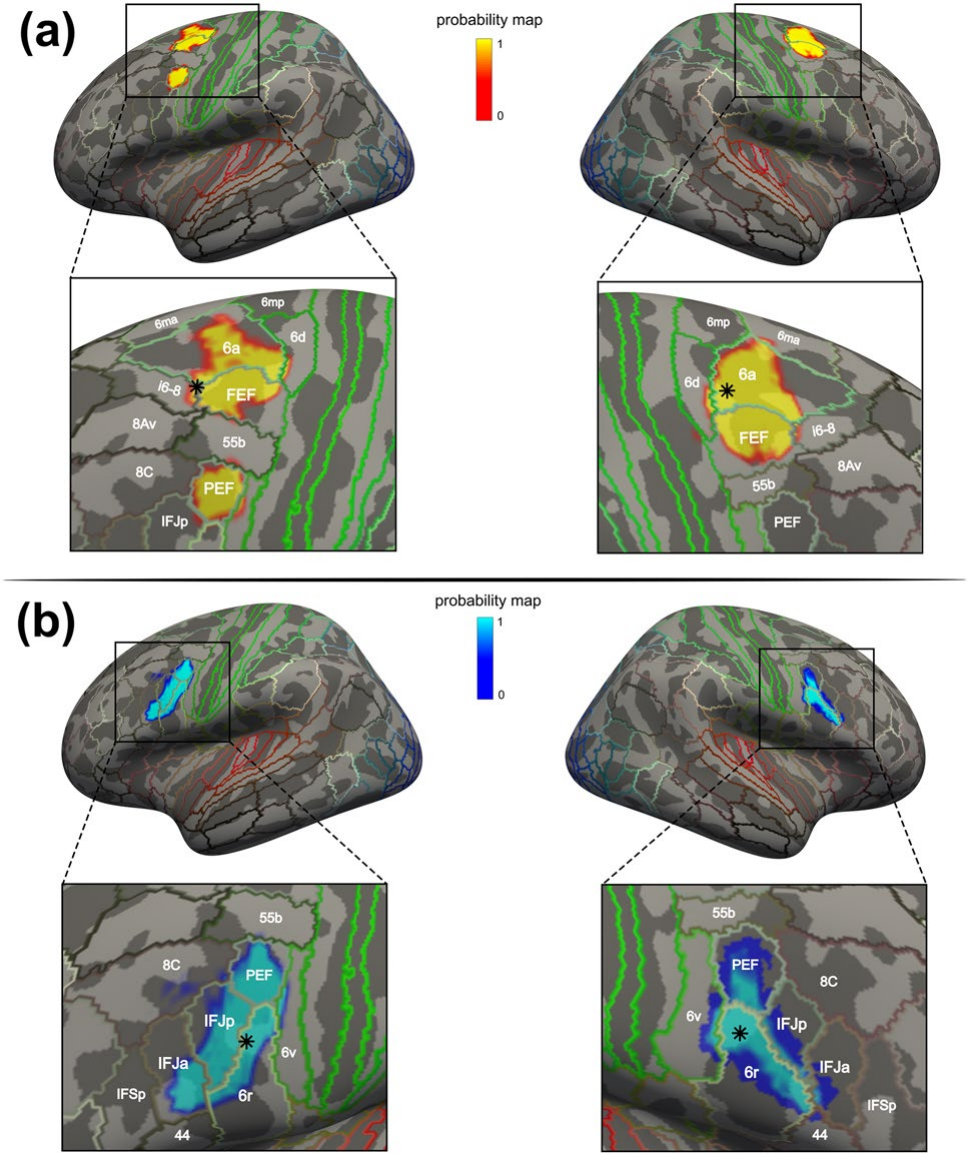
Projection on FSaverage of the FEF and IFJ main clusters and comparison with the MMP1. **A** Vertices corresponding to the FEF clusters. Both clusters covered the middle and rostral parts of the FEF label as defined according to the MMP1 atlas, but they also covered large parts of area 6a. In the LH, vertices were also localized in the iFEF, which matches almost exactly the boundaries of area PEF from the atlas. The LH FEF ALE peak was localized within area i6-8, just anterior to the FEF, and the RH FEF ALE peak was localized within area 6a, dorsal to FEF. Despite this difference, both peaks were localized near the junction of the sPCS and the SFS, in the anterior bank and the posterior banks of the sPCS, respectively. **B** Vertices corresponding to the IFJ clusters. They showed a similar elongated shape that approximately followed the posterior iPCS and encroached onto the IFS, and they spanned multiple MMP1 areas. Importantly, we found that in both hemispheres the IFJ ALE peaks were localized near the junction of the iPCS and the SFS within area 6r, posteriorly to the IFJp

Finally, in our opinion, the most interesting results of these comparisons were those obtained from the projection of our main FEF and IFJ clusters on the FSaverage surface using the method from Wu et al. (2018) where we could carefully examine their spatial relationship with the MMP1 (see Fig. 4). The FEF clusters covered almost the entire middle and anterior part of the FEF (as defined by the corresponding MMP1 label) but also large parts of the middle and posterior 6a region. Moreover, the left and right hemisphere ALE peaks were found within area i6-8 and area 6a, anteriorly and dorsally relative to the FEF, respectively. The IFJ clusters instead spanned multiple MMP1 labels, including areas PEF, 6r, IFJp and IFJa. While in the left hemisphere, the majority of the vertices of the cluster were localized in the middle and posterior aspect of the IFJp, in the right hemisphere most of the vertices were localized in the ventral 6r region. Crucially, in both hemispheres, however, the ALE peaks were localized in the latter region, ventral to the IFJp.

### Meta-analytic connectivity modeling results

**Fig. 5:**
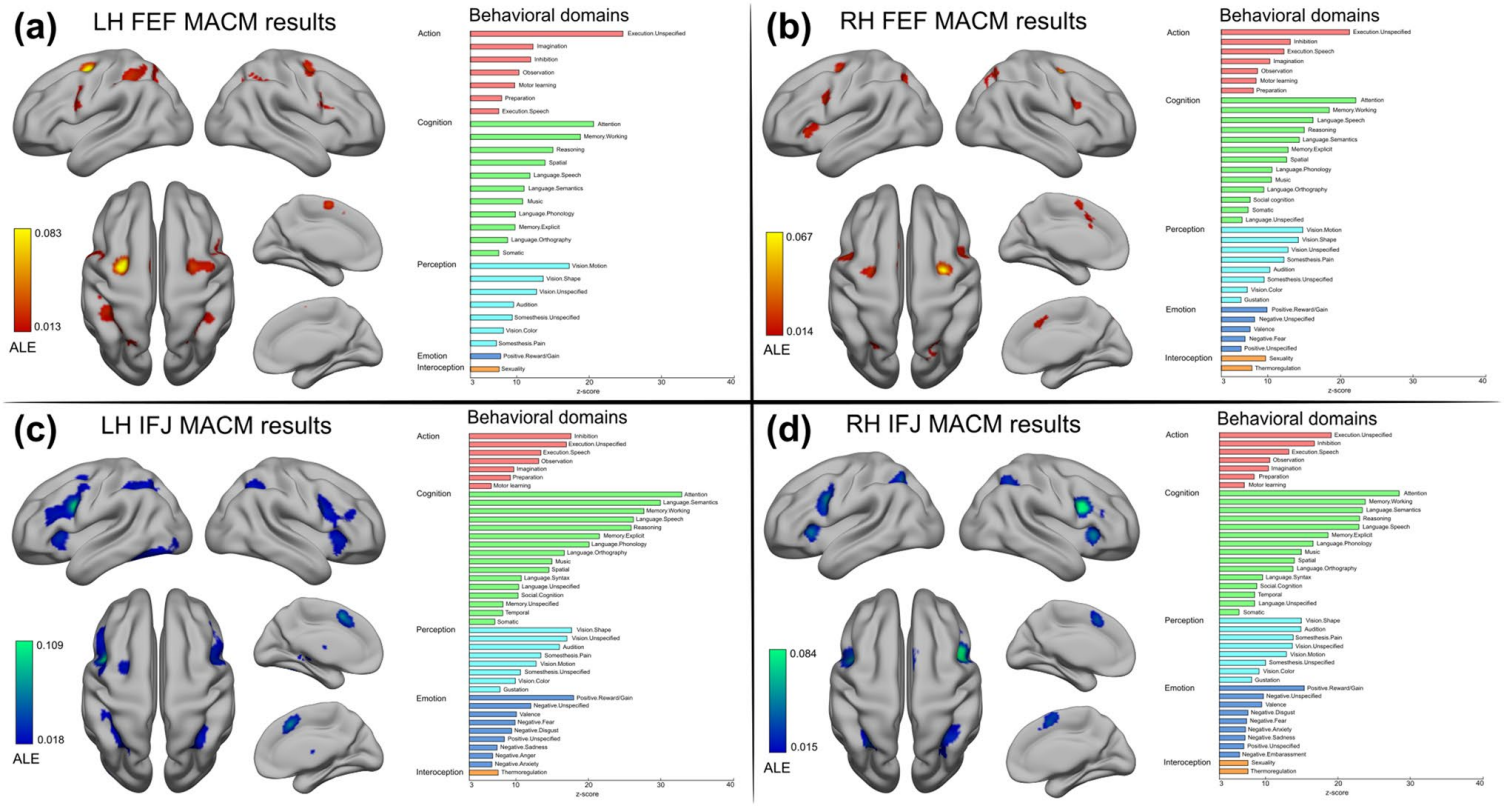
Meta-analytic connectivity modeling results. **A** and **B** depict the coactivation profiles of the FEF, and **C** and **D** depict the coactivation profiles of the IFJ. On the right side of each panel: decoding results of the significant associations with behavioral domains (*p* < 0.05, Bonferroni corrected)

The MACM analysis of the FEF and IFJ revealed a broad set of regions that coactivated with these seeds in the BrainMap database encompassing the frontal, parietal and temporal cortices (see Fig. 5). The LH FEF seed coactivated with six other clusters (see Fig. 5A), and the RH FEF coactivated with eight other clusters (Fig. 5B). Interestingly, while these FEF coactivations included as expected medial oculomotor regions (the SCEF) and the SPL/IPL, in both analyses we found coactivated clusters in the bilateral ventral PFC, which included parts of the iFEF and the IFJ based on their localization relative to the iPCS and the IFS. The LH IFJ coactivated with a broad set of other nine clusters (Fig. 5C), and the RH IFJ coactivated with five other clusters (Fig. 5D). The coactivations of these bilateral seeds spread onto the IFS and ventrally in the insular cortex and claustrum. Again, these coactivations included clusters in the SCEF, the SPL/IPL and the angular gyrus. In contrast to the FEF coactivations, where the bilateral IFJ was always coactivated, we did not find FEF coactivations in the IFJ MACM results, except for the ipsilateral FEF in the LH IFJ MACM analysis. Another crucial difference was that in this analysis, we found a large cluster in the left temporal lobe that included the fusiform gyrus and the inferior occipital cortex. We confirmed these patterns by performing an ALE contrast analysis between the FEF and IFJ MACM results in each hemisphere (see Supplementary Information p. 23). To summarize, while the FEF MACM analysis showed that this region is consistently coactivated with the ventrolateral PFC and regions in the posterior parietal cortex across paradigms, the IFJ had more widespread coactivation patterns (particularly in the LH), being more strongly connected with the rest of the PFC and with the insular cortex and claustrum, and possessing a differential connectivity pattern with regions of the inferior temporal cortex. In contrast, we found differential coactivations of the LH and RH FEF with the ipsilateral precuneus/SPL and lateral intraparietal areas. In addition to revealing the task-based functional connectivity fingerprint of these regions, our functional decoding approach also allowed us to uncover the behavioral domains significantly associated with each of them (Fig. 5, right side of each panel; see Supplementary Information p. 23 for a summary of these results).

## Discussion

The PFC is essential to several aspects of flexible goal-driven behavior that are mediated by specialized brain regions (Fuster 2001; Miller and Cohen 2001; O’Reilly 2010). The FEF and the IFJ have been primarily implicated in covert spatial attention and oculomotor control on the one hand (Fiebelkorn and Kastner 2020; Vernet et al. 2014), and attention, working memory, and cognitive control on the other (Bedini and Baldauf 2021; Brass et al. 2005). Their localization has been traditionally associated with the major sulci of the posterior-lateral PFC, namely the SFS and the sPCS, and the iPCS and the IFS, respectively (Amiez et al. 2006; Derrfuss et al. 2009). Due to the large body of empirical work that has accumulated over the past years on these regions (Bedini and Baldauf 2021) and the parallel development of more robust meta-analytic techniques for neuroimaging data (Fox et al. 2014), we felt the need to re-examine the previous results in light of the current evidence, with a specific focus on overcoming discrepancies in the definition and localization of these regions using fMRI in humans. In particular, in this study, we sought to accurately estimate the precise localization of these regions in standard space by performing a coordinate-based meta-analysis using the ALE technique (Eickhoff et al. 2012). To model the spatial convergence of activations within the FEF, we analyzed data from 35 fMRI studies (35 experiments) that investigated the planning and execution of prosaccades and antisaccades contrasted against a fixation baseline in 449 subjects. To model the spatial convergence within the IFJ, we analyzed data from 30 fMRI studies (32 experiments) that investigated visual attention, working memory, and cognitive control, in 563 subjects. In contrast to previous ALE meta-analyses that relied on the false discovery rate (Derrfuss et al. 2005; Grosbras et al. 2005; Jamadar et al. 2013), our study implemented a much more conservative method for multiple comparisons correction, namely voxel-level FWE, following the recommendations of Eickhoff et al. (2016). We also included only higher field fMRI studies (3 T and 4 T) to make our inference spatially more reliable. Moreover, testing for significance in each voxel individually enabled us to carry out a fine-grained assessment of activations across experiments. Crucially, we found that by modeling activity across studies (thus partially overcoming inter-individual variability), sulcal landmarks are indeed consistently associated with both regions, as indicated by the location of the ALE peak values (see Fig. 1 and Table 3). Our results thus suggest a robust association of structure and function in these higher PFC regions (Frost and Goebel 2012; Miller et al. 2021; Van Essen 2007; Wang et al. 2015), analogous to what previous studies have shown in early visual regions (Benson et al. 2012; Hinds et al. 2008). We suggest that this association should be examined by future fMRI studies more systematically at the individual-subject level (Amiez et al. 2006; Amiez and Petrides 2018; Derrfuss et al. 2009, 2012). Given the limitations of previous meta-analyses (Derrfuss et al. 2005; Grosbras et al. 2005; Jamadar et al. 2013; Paus 1996), we recommend using the coordinates reported in the present study to define the FEF and IFJ based on the ALE technique.

### FEF localization in standard space

The FEF is arguably one of the most important regions of a network involved in the planning and execution of saccadic eye movements and has been extensively studied in primates (Schall et al. 2020; Tehovnik et al. 2000). In humans, this network comprises a set of regions in the lateral and medial frontal cortex, posterior parietal cortex, and subcortical nuclei (Corbetta et al. 1998; Grosbras et al. 2005), and has been mainly investigated with fMRI over the past 25 years, enabling to characterize their respective functions (Coiner et al. 2019; McDowell et al. 2008). Following the crucial foundation set by non-human primate neurophysiology (Bruce et al. 1985; Buschman and Miller 2009; Moore and Fallah 2001), the human FEF has not only been implicated in visually guided and voluntary saccades, and other oculomotor behaviors (Vernet et al. 2014), but also in covert shifts of spatial attention, spatial working memory, and also more complex executive functions (Fiebelkorn and Kastner 2020; Vernet et al. 2014). Converging lines of research suggest that the FEF acts as a spatial priority map (Fiebelkorn and Kastner 2020; Jerde et al. 2012; Sprague and Serences 2013). The localization of the human FEF is, however, highly debated and affected by strong spatial variability (Bedini and Baldauf 2021; Vernet et al. 2014), possibly due to inter-individual differences that are obscured when reporting group-level results. Previous ALE meta-analyses provided evidence of consistent activations within FEF across PET and fMRI experiments investigating prosaccades and antisaccades (Cieslik et al. 2016; Grosbras et al. 2005; Jamadar et al. 2013). However, given the coarser spatial resolution of PET and low-field strength MRI scanners (i.e., 1.5 T) and acquisition methods that were included in these previous meta-analyses, these studies were only partially able to accurately infer spatial convergence across experiments, as well as dissociations across paradigms and contrasts. In addition, some of these studies relied on earlier implementations of the ALE technique, which allowed for within-study effects (Turkeltaub et al. 2012), and critically, more liberal statistical thresholds and multiple comparisons correction methods (Eickhoff et al. 2016). Finally, another aspect that is difficult to evaluate retrospectively was the finding of two errors in the multiple comparisons correction step in earlier implementations of GingerALE (Eickhoff et al. 2017), which presumably affected the meta-analyses published prior to that report. In this study, we attempted to overcome some of these limitations by applying a conservative multiple comparisons correction method and by including higher field fMRI studies (i.e., 3 T or 4 T) to accurately infer the localization of the human FEF in standard space. Our ALE results, obtained by analyzing prosaccades > fixation, antisaccades > fixation, and prosaccades and antisaccades > fixation contrasts across 35 fMRI experiments (see Table S1), show that the highest spatial convergence based on the ALE value was found within two bilateral clusters in the dorsolateral PFC, localized in the anterior bank of the sPCS, near its junction with the SFS (see Fig. 1; Table 2). The comparison with one of the most comprehensive brain parcellations available to date (the MMP1 by Glasser et al. 2016) revealed some interesting spatial relationships with our results. We found that the FEF ALE peaks did not match the corresponding label from the atlas, suggesting that there may be important differences in the way the FEF is defined across methods. The MMP1 was created by a careful semi-manual delineation combining structural MRI (cortical thickness and myelin ratio), resting-state fMRI connectivity, and retinotopic mapping techniques (Glasser et al. 2016). Additionally, fMRI contrasts from nine tasks were also employed to infer areal boundaries, which were chosen to optimally balance breadth vs depth and scan time (Elam et al. 2021). Although we regard the MMP1 as a step change in our understanding of brain organization, and of the fine-grained organization and structure of the PFC in humans and non-human primates (Donahue et al. 2018), we would like to suggest that more information gathered from task-based fMRI will be needed to better understand the functional subdivision of the posterior-lateral PFC. More specifically, following the labeling scheme proposed by the MMP1, major efforts should be made to isolate FEF activity from posterior activity in the premotor cortex on the one hand (area 6d), and from a newly discovered language selective region (area 55b) that borders the FEF ventrally on the other (Glasser et al. 2016). Ultimately, future developments of a functional localization method will facilitate the convergence of atlas-based and meta-analytic fMRI information to allow the delineation of anatomical clusters of activation within FEF with adequate functional specificity.

In this direction, the seminal fMRI study by Mackey et al. (2017) identified three distinct visual field maps in the PFC localized in the sPCS (sPCS1 and sPCS2) and the iPCS. By examining the correspondences between their results and the MMP1 (see Fig. 8 from their study), they found that the sPCS2 corresponded to the FEF, while the sPCS1 corresponded to areas 6a and 6d. Interestingly, they also reported that in all subjects and both hemispheres, the foveal representation was localized in the fundus of the sPCS, at its intersection with the SFS. This description closely matches the localization of our ALE peaks, which raises the question of whether the fMRI contrasts we included in the present meta-analysis could be targeting specific neural populations within the FEF. It is well established that in the macaque, a population of neurons shows increased firing rates when the animal is fixating and is inhibited when executing saccades (hence termed ‘fixation’ neurons; Hanes et al. 1998; Lowe and Schall 2018). Are these neural populations also present in humans, and how are they distributed within the FEF? What is the role of saccadic amplitude in isolating peaks of activity within the FEF (see Grosbras 2016)? An additional aspect that may be worth investigating is whether the activations found in one or more of these clusters (for example, the iFEF) are dependent on some artifacts present in the experimental design or analysis. In the 35 experiments we analyzed, 10 did not record eye movements in the scanner (see Table S1), leaving open the possibility that some of these clusters may have also been driven by spurious neural activity that was not exclusively related to saccadic behavior. It is well documented that eye blinks can contaminate BOLD signal (Bristow et al. 2005; Hupé et al. 2012), and this fact was invoked to explain discrepancies in the oculomotor organization in primates (Tehovnik et al. 2000) and as a signal driving iFEF responses (Amiez and Petrides 2009; Kato and Miyauchi 2003). In conclusion, we strongly agree with the general caveat that the way the FEF is defined is ultimately constrained by the technique employed (Schall et al. 2020; Vernet et al. 2014), and in particular its spatial resolution. The localization and the extent of the FEF cluster should be inferred based on the convergence of multiple criteria (primarily architectonic, sulcal, functional, connectional, and also comparative). In the present study, we provided a standard using ALE, which we suggest can lead to clarifying how inter-individual variability affects FEF localization and its relationship to sulcal organization (Amiez et al. 2006; Glasser et al. 2016). Refinements in the careful mapping of the human FEF will be essential to bridge research in humans and non-human primates and for testing hypotheses about homologies in the organization of the PFC across species, for example, based on connectivity information (Hutchison et al. 2012; Mars et al. 2021; Neggers et al. 2015; Sallet et al. 2013).

### IFJ localization in standard space

The study of the role of the ventrolateral PFC in various cognitive functions such as visual attention, working memory, and cognitive control led to the definition of the IFJ as a separate brain region involved in critical aspects of all these functions (Baldauf and Desimone 2014; Brass et al. 2005; Derrfuss et al. 2005; Zanto et al. 2010). This region appears to be tightly coupled with specific sulcal landmarks (Derrfuss et al. 2009) and belongs to the frontoparietal network (Cole and Schneider 2007; Yeo et al. 2011). In this study, we pooled together results from the various tasks that have been used to localize this region (see Table S2) ranging from attentional (i.e., RSVP/oddball; Asplund et al. 2010; and endogenous cueing paradigms; Baldauf and Desimone 2014; Zhang et al. 2018), working memory (primarily n-back paradigms; Rottschy et al. 2012; Zanto et al. 2010), and cognitive control paradigms (i.e., task-switching and Stroop tasks; Derrfuss et al. 2012). Following a previous ALE meta-analysis (Derrfuss et al. 2005), we reasoned that the spatial convergence across these paradigms (rather than a single task and/or contrast) would allow us to accurately infer the localization of the IFJ in standard space. Consistent with our hypothesis, we found two prominent clusters of activation in the ventrolateral PFC. Based on the ALE peak values, the highest convergence was found in the posterior bank of the iPCS, approximately at the height of its ventral junction with the IFS (see Fig. 3; Table 2). The comparison of these results with the MMP1 revealed additional interesting topographic differences. The IFJ ALE peaks were localized just ventral and slightly posterior to the corresponding labels from the atlas (IFJa and IFJp), and the ALE clusters included vertices from several other brain regions. These results suggest that many of the paradigms that target the IFJ will also tend to involve adjacent multiple-demand regions, which may conceal its exact boundaries.

An interesting approach combining different paradigms and using a conjunction analysis to localize the IFJ was presented by Stiers and Goulas (2018), which may overcome some of the previous limitations. The authors analyzed the voxel responses across three different tasks (Eriksen flanker task, back matching or n-back task, and a response scheme switching task) to define the prefrontal nodes of the multiple-demand system in 12 subjects. A manipulation of task difficulty in each of the previous tasks was used to identify voxels that were modulated by increasing cognitive demands, which were used to define ROIs in each subject in a conjunction analysis across tasks for further analyzing their relative task preference and functional connectivity patterns. This analysis revealed local maxima of activity within the IFJ, where voxels with different task preferences exhibited distinct functional connectivity patterns with the rest of the brain (Stiers and Goulas 2018). Based on these results, it may be argued that no single task alone would adequately capture the selectivity patterns of neural populations within the IFJ; rather, manipulations of task difficulty combined with the administration of different paradigms could provide an unbiased way of localizing this region in individual participants. The present study provides a standard using ALE, which should help further understand how inter-individual variability affects IFJ localization and its relationship to sulcal organization (Derrfuss et al. 2009). Future meta-analyses should better clarify how activations from different tasks that tap cognitive control functions map onto the ventro-lateral PFC and assess whether they may recruit distinct subregions near the IFJ (Nee et al. 2013), which was, however, outside the scope of our present work (see Rodríguez-Nieto et al. 2022 for a comprehensive meta-analysis).

### Whole-brain coactivation patterns of the FEF and IFJ

An additional goal of the present study was to uncover the task-based functional connectivity fingerprint of the FEF and the IFJ in a data-driven fashion. We retrieved from the BrainMap database all the studies that reported activations within a cuboid seed centered around the FEF and IFJ standard coordinates found in our ALE main localizer analyses and we employed the MACM technique to uncover their coactivation profiles (Langner and Camilleri 2021). Importantly, while previous studies already performed MACM analyses on the FEF (Cieslik et al. 2016; Genon et al. 2017) and the IFJ (Muhle-Karbe et al. 2016; Sundermann and Pfleiderer 2012), our study is to our knowledge the first that used this technique on the results of an ALE analysis specifically aimed at localizing these regions (and not a manual or atlas-based delineation) using a narrow seed extent (6 mm). Our MACM analysis allowed adequately powered inference in each seed region (Eickhoff et al. 2016) and revealed broad networks of coactivations that encompassed the frontal, parietal and temporal cortices (see Fig. 5). The most remarkable differences between FEF and IFJ coactivation patterns were that on the one hand, the LH FEF and RH FEF coactivated with the bilateral ventrolateral PFC (iFEF and IFJ), whereas only the LH IFJ coactivated with the LH FEF in the experiments retrieved. On the other hand, the LH IFJ had stronger and more widespread coactivations in PFC and the insular cortex and was also coactivated with the inferotemporal cortex. These coactivation patterns may be essential for the IFJ to perform its role in feature- and object-based attention tasks (Baldauf and Desimone 2014; De Vries et al. 2021; Liu et al. 2011; Liu 2016; Meyyappan et al. 2021; Soyuhos and Baldauf 2023; Zhang et al. 2018) and could be in turn supported by its underlying anatomical connectivity patterns (Baldauf and Desimone 2014; Bedini et al. 2021). These results are consistent with the idea of a dorso-ventral segregation of frontoparietal coactivations forming a ‘spatial/motor’ and a ‘non-spatial/motor network’, which are in turn associated with the first and third branch of the superior longitudinal fasciculus, respectively (Parlatini et al. 2017). In addition, the MACM contrast between the coactivation patterns of FEF and IFJ fit very well with a recent report of these regions’ functional connectivity in source-reconstructed resting-state MEG data, which showed stronger connectivity of the FEF with the dorsal ‘where’ visual pathway (especially in the alpha and beta bands), and stronger connectivity of the IFJ with the ventral ‘what’ visual pathway (especially in the delta and gamma bands; Soyuhos and Baldauf 2023). Finally, our functional decoding results suggest that these systematic coactivation patterns allow these regions to support multiple yet specialized roles in flexible goal-driven behavior (Assem et al. 2021; Genon et al. 2017; Muhle-Karbe et al. 2016; Ngo et al. 2019; Ruland et al. 2022).

## Conclusion

Our study provides the accurate localization of two regions of the posterior-lateral PFC, namely the FEF and the IFJ. These regions are tightly coupled with sulcal landmarks as measured using fMRI across over 400 and 500 subjects, respectively, with the FEF being localized at the junction of the sPCS and the SFS, and the IFJ at the junction of the iPCS and the IFS. Functionally, they appear to be organized according to a dorso-ventral gradient, going from areas responsible for sensorimotor transformations and action execution (FEF, iFEF), to areas that are involved in maintaining and updating behavioral goals according to internal representations (IFJ; Abdallah et al. 2022; Nee et al. 2013; O’Reilly 2010). Taken together, our findings aim at proposing a consensus localization of these regions in standard space, and meta-analytic groundwork to investigate the relationship between functional specialization and connectivity in large publicly available neuroimaging datasets (e.g., Markiewicz et al. 2021; Van Essen et al. 2013), as well as to guide future non-invasive brain stimulation studies.

## Supplementary Information

Below is the link to the electronic supplementary material.

Supplementary file1 (PDF 13401 KB)

## Data Availability

The FEF and IFJ localizer sample ALE results are available in NeuroVault at: https://neurovault.org/collections/KLNRWMMU/. All the other results can be made available upon request from the corresponding author.
