# Supplementary material for: Accurate localization and coactivation profiles of the frontal eye field and inferior frontal junction: an ALE and MACM fMRI meta-analysis
Source: Brain Struct Funct. 2023 Apr 24;228(3-4):997–1017. doi: 10.1007/s00429-023-02641-y (PMC10147761; doi:10.1007/s00429-023-02641-y)
Supplement: Supplementary file 1 — Supplementary file1 (PDF 13401 KB) [file 429_2023_2641_MOESM1_ESM.pdf]

Accurate localization and coactivation profiles of the Frontal Eye Field and Inferior Frontal Junction: an ALE and MACM fMRI meta-analysis

Marco Bedini[1,3*], Emanuele Olivetti[1,2], Paolo Avesani[1,2] & Daniel Baldauf[1]

1. Center for Mind/Brain Sciences (CIMeC), University of Trento, Trento, Italy

2. NeuroInformatics Laboratory (NILab), Bruno Kessler Foundation (FBK), Trento, Italy

3. Department of Psychology, University of California, San Diego, La Jolla, California, USA

* Corresponding author at: Center for Mind/Brain Sciences, University of Trento
via delle Regole, 101, 38123, Trento (TN), Italy
ORCID: 0000-0002-2018-5175
E-mail: marco.bedini@unitn.it

Keywords: Prefrontal Cortex, Saccades, Working memory, Cognitive Control, Activation Likelihood Estimation, Meta-analytic Connectivity Modeling

SUPPLEMENTARY INFORMATION

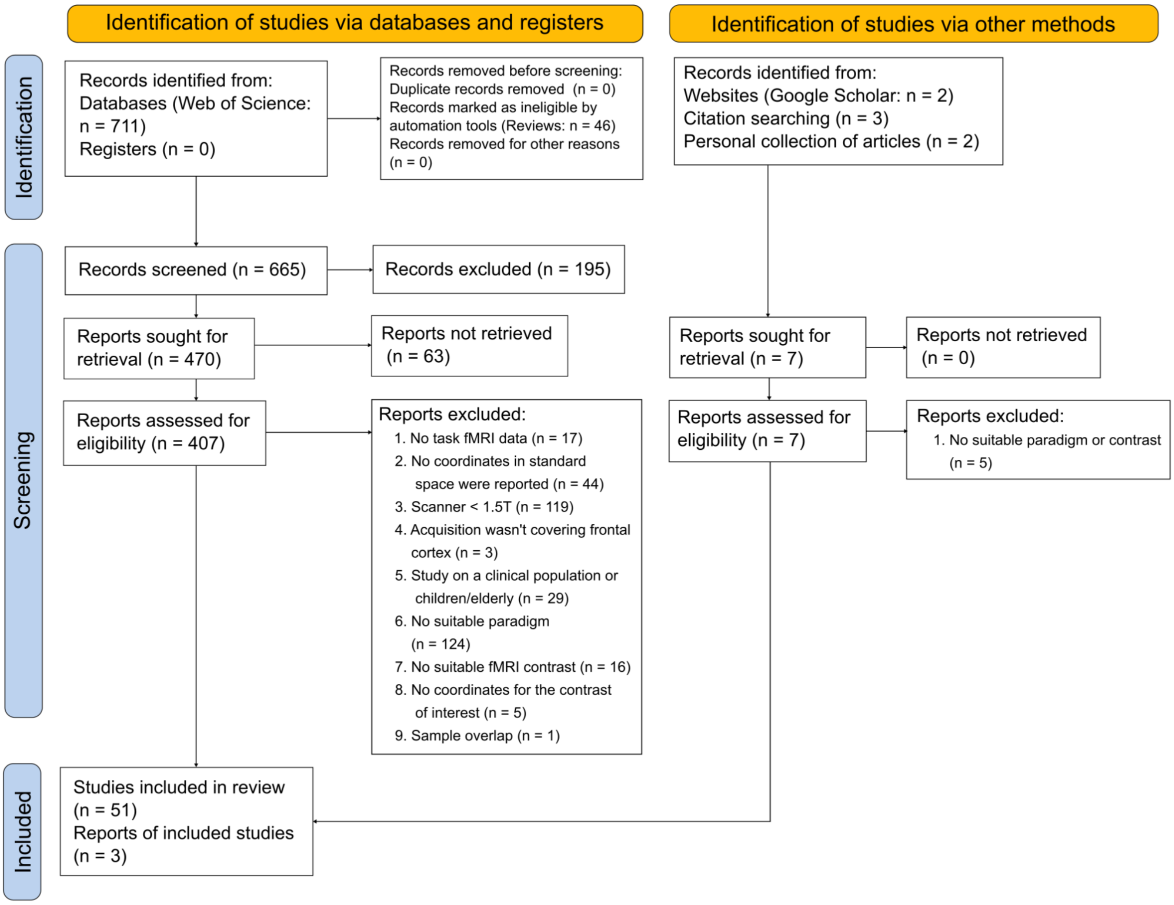

FIGURE S1 PRISMA flow chart FEF sample

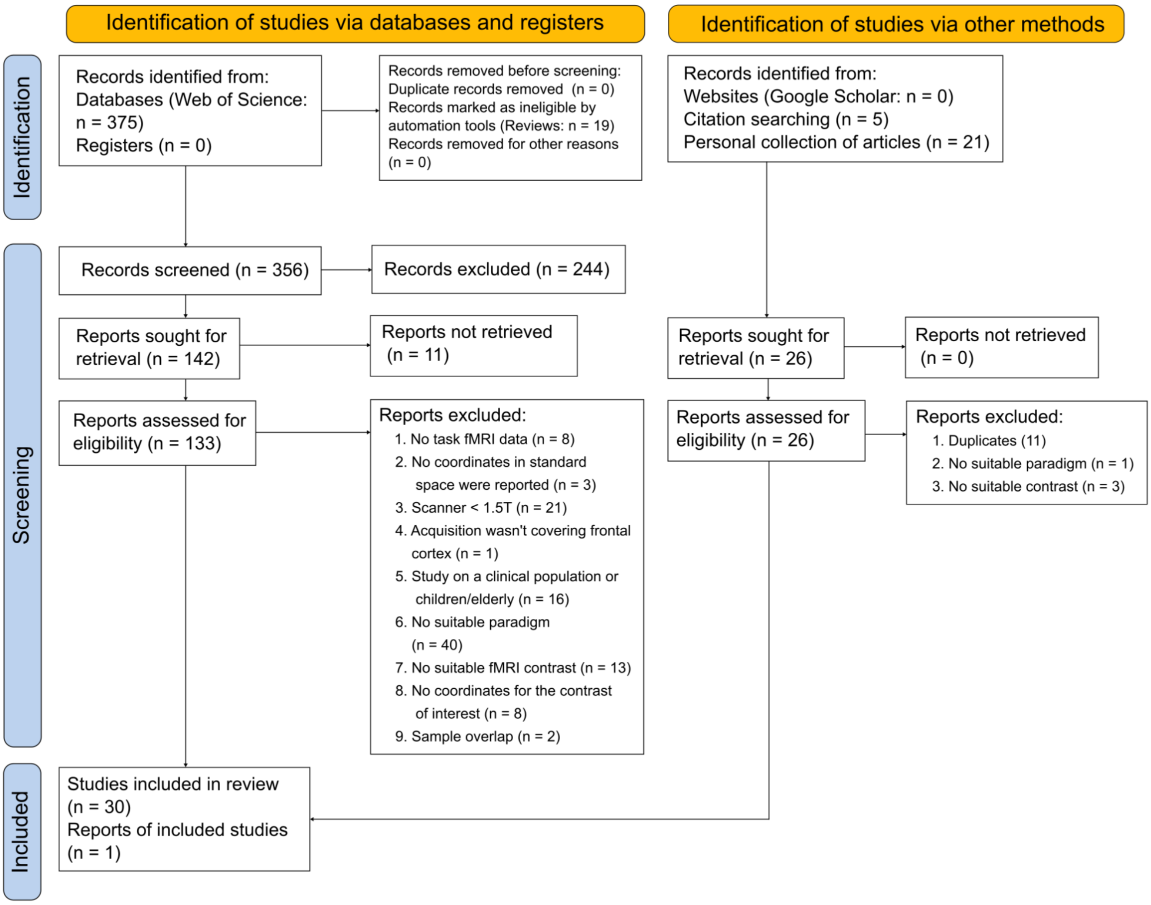

FIGURE S2 PRISMA flow chart IFJ sample

Supplementary Table 1 List of the studies included in the FEF localizer sample

Study	N	Age	Paradigm	Contrast	Scanner	Design	Eye tracker
Alkan et al. (2011)	8	26 ± 4	Oculomotor task	Prosaccades > Fixation	3T	Blocked	Y
Amiez and Petrides (2018)	13	22.6 ± 2.8	Oculomotor task	Prosaccades > Fixation	3T	Blocked	N
Atmaca et al. (2013)	11	26.9; 22-33	Oculomotor task	Prosaccades > Fixation	3T	Blocked	N
Bär et al. (2016)	14	22-56	Oculomotor task	Prosaccades > Fixation	3T	Blocked	N
Bär et al. (2016)	14	22-56	Oculomotor task	Antisaccades > Fixation	3T	Blocked	N
Berman et al. (1999)	11	25.6 ± 7.1; 18-43	Oculomotor task	Prosaccades > Fixation	3T	Blocked	N
Braga et al. (2016)	20	26.2; 21-36	Oculomotor task	Prosaccades > Fixation	3T	Blocked	Y
Brown et al. (2006)	10	26; 22-33	Oculomotor task	Prosaccades > Fixation	4T	*Event-related*	Y
Brown et al. (2006)	10	26; 22-33	Oculomotor task	Antisaccades > Fixation	4T	*Event-related*	Y
Connolly et al. (2000)	7	24.8 ± 3.2	Oculomotor task	Prosaccades > Fixation	4T	Blocked	N
Connolly et al. (2000)	7	24.8 ± 3.2	Oculomotor task	Antisaccades > Fixation	4T	Blocked	N
Connolly et al. (2002)	8	NA	Oculomotor task	Prosaccades > Fixation	4T	Blocked	N
Connolly et al. (2005)	5	NA	Oculomotor task	Prosaccades > Fixation	4T	Blocked	Y
Connolly et al. (2007)	8	NA	Oculomotor task	Prosaccades > Fixation	4T	Blocked	N
Christophel et al. (2018)	22	24.4 ± 0.83	Oculomotor task	Prosaccades > Fixation	3T	*Event-related*	N
Curtis & Connolly (2008)	12	21-35	Oculomotor task	Pro- & Antisaccades > Fixation	3T	*Event-related*	Y
DeSouza et al. (2003)	10	26.6 ± 1.0	Oculomotor task	Pro- & Antisaccades > Fixation	4T	Blocked	Y
Duecker et al. (2013)	20	19-28	Oculomotor task	Prosaccades > Fixation	3T	Blocked	N
Fernandez-Ruiz et al. (2018)	25	21.7 ± 1.9; 18-25	Oculomotor task	Pro- & Antisaccades > Fixation	3T	*Event-related*	Y
Frannson et al. (2014)	24	26.7 ± 4.9	Oculomotor task	Prosaccades > Fixation	3T	Blocked	Y
Furlan et al. (2016)	6	NA	Oculomotor task	Pro- & Antisaccades > Fixation	3T	*Event-related*	Y
Guo et al. (2012)	12	19-31	Oculomotor task	Prosaccades > Fixation	3T	Blocked	Y
Gurel et al. (2018)	16	23.2	Oculomotor task	Prosaccades > Fixation	3T	Blocked	Y
Heinen et al. (2006)	5	20-37	Oculomotor task	Prosaccades > Fixation	3T	Blocked	Y
Hubl et al. (2008)	7	31 ± 9	Oculomotor task	Prosaccades > Fixation	3T	Blocked	N
Jamadar et al. (2015)	23	25.8; 18-43	Oculomotor task	Prosaccades > Fixation	3T	*Event-related*	Y
Jamadar et al. (2015)	23	25.8; 18-43	Oculomotor task	Antisaccades > Fixation	3T	*Event-related*	Y
Jarvstad & Gilchrist (2019)	23	NA	Oculomotor task	Prosaccades > Fixation	3T	Blocked	Y
Kastner et al. (2007)	4	20-36	Oculomotor task	Prosaccades > Fixation	3T	Blocked	Y
Kurata et al. (2005)	6	29-38	Oculomotor task	Prosaccades > Fixation	3T	Blocked	N
Levy et al. (2007)	4	24-43	Oculomotor task	Prosaccades > Fixation	3T	Blocked	N
Neggers et al. (2007)	15	NA	Oculomotor task	Pro- & Antisaccades > Fixation	3T	Blocked	N
Pierce et al. (2019)	30	25.7 ± 4.1	Oculomotor task	Prosaccades > Fixation	3T	Blocked	Y
Schon et al. (2008)	17	21.29 ± 3.72	Oculomotor task	Prosaccades > Fixation	3T	Blocked	N
Schwerdtfeger et al. (2013)	14	29.6 ± 9.6	Oculomotor task	Pro- & Antisaccades > Fixation	3T	*Event-related*	Y
Tamber-Rosenau et al. (2018)	8	28.5 ± 3.3	Oculomotor task	Prosaccades > Fixation	3T	Blocked	Y
Tark & Curtis (2009)	5	22-39	Oculomotor task	Prosaccades > Fixation	3T	Blocked	Y
Tibber et al. (2010)	16	21-40	Oculomotor task	Prosaccades > Fixation	3T	Blocked	Y
Tu et al. (2006)	10	27.9 ± 3.18	Oculomotor task	Antisaccades > Fixation	3T	Blocked	N

Supplementary Table 1 (Continued)

Study	Independent fMRI localizer	Field of View (FOV)	Results reported for	N° of foci	Multiple FEF foci	Software	Space
Alkan et al. (2011)	N	220 mm	Whole-brain	19	N	AFNI	Talairach
Amiez and Petrides (2018)	N	NA	FEF foci	2	N	SPM12b	MNI
Atmaca et al. (2013)	Y	192 mm	Whole-brain	24	Y	SPM8	MNI
Bär et al. (2016)	N	240 mm	Whole-brain	12	N	BrainVoyager QX 1.10	Talairach
Bär et al. (2016)	N	240 mm	Whole-brain	18	Y	BrainVoyager QX 1.10	Talairach
Berman et al. (1999)	N	40 x 20 mm	ROIs	14	Y	AFNI	Talairach
Braga et al. (2016)	Y	220 mm	Whole-brain	7	N	FSL	MNI
Brown et al. (2006)	N	220 mm	Whole-brain	17	N	BrainVoyager 2000	Talairach
Brown et al. (2006)	N	220 mm	Whole-brain	21	N	BrainVoyager 2000	Talairach
Connolly et al. (2000)	N	192 mm	Whole-brain	7	Y	Stimulate	Talairach
Connolly et al. (2000)	N	192 mm	Whole-brain	13	Y	Stimulate	Talairach
Connolly et al. (2002)	Y	192 mm	ROIs	4	N	Stimulate	Talairach
Connolly et al. (2005)	Y	192 mm	ROIs	5	N	Stimulate / BrainVoyager 4.9	Talairach
Connolly et al. (2007)	Y	192 mm	ROIs	2	Y	Stimulate / BrainVoyager 4.9	Talairach
Christophel et al. (2018)	Y	192 mm	FEF foci	2	N	SPM8	MNI
Curtis & Connolly (2008)	N	192 mm	ROIs	32	Y	Caret	MNI
DeSouza et al. (2003)	Y	192 mm	Whole-brain	9	N	BrainVoyager 2000 4.4	Talairach
Duecker et al. (2013)	Y	192 mm	FEF foci	2	N	BrainVoyager QX 2.3	Talairach
Fernandez-Ruiz et al. (2018)	N	211 mm	Whole-brain	18	N	BrainVoyager QX 2.8.4	Talairach
Fransson et al. (2014)	N	288 mm	Whole-brain	8	N	SPM8	MNI
Furlan et al. (2016)	N	192 mm	ROIs	6	N	BrainVoyager QX 2.3	Talairach
Guo et al. (2012)	Y	192 mm	ROIs	6	N	SPM8	Talairach
Gurel et al. (2018)	Y	192 mm	FEF foci	2	N	BrainVoyager QX	Talairach
Heinen et al. (2006)	Y	NA	ROIs	4	N	VISTASOFT	Talairach
Hubl et al. (2008)	Y	192 mm	Whole-brain	9	N	BrainVoyager QX	Talairach
Jamadar et al. (2015)	Y	192 mm	Whole-brain	47	N	SPM8	MNI
Jamadar et al. (2015)	Y	192 mm	Whole-brain	47	N	SPM8	MNI
Jarvstad & Gilchrist (2019)	N	192 mm	Whole-brain	4	N	FSL v.5.06-1	MNI
Kastner et al. (2007)	Y	256 mm	ROIs	5	N	AFNI	Talairach
Kurata et al. (2005)	N	220 mm	Whole-brain	14	N	Advanced Visual Systems & AFNI	Talairach
Levy et al. (2007)	Y	192 mm	ROIs	5	N	BrainVoyager QX	Talairach
Neggers et al. (2007)	Y	224 x 256 x 128 mm	ROIs	4	Y	SPM2	MNI
Pierce et al. (2019)	Y	NA	Whole-brain	9	Y	SPM12	MNI
Schon et al. (2008)	Y	200 mm	Whole-brain	19	N	SPM2	MNI
Schwerdtfeger et al. (2013)	N	211 x 211 mm	ROIs	10	Y	BrainVoyager QX 1.9	Talairach
Tamber-Rosenau et al. (2018)	Y	240 x 240 mm	ROIs	6	N	BrainVoyager QX v.1.10.2–2.8	Talairach
Tark & Curtis (2009)	Y	192 mm	FEF foci	2	N	Caret	MNI
Tibber et al. (2010)	Y	192 mm	ROIs	9	N	SPM5	Talairach
Tu et al. (2006)	Y	250 x 250 mm	Whole-brain	21	N	SPM2	MNI

Supplementary Table 2 List of the studies included in the IFJ localizer sample

Study	N	Age	Paradigm	Contrast	Scanner	Design	Eye tracker
Armbruster et al. (2012)	20	23.5; 20–32	Task-switching paradigm	Task switch > Distractor trials	3T	*Event-related*	N
Armbruster et al. (2012)	20	23.5; 20–32	Task-switching paradigm	Distractor inhibition > Baseline	3T	*Event-related*	N
Armbruster et al. (2012)	20	23.5; 20–32	Task-switching paradigm	Task switch > Repetition trials	3T	*Event-related*	N
Asplund et al. (2010)	30	NA	RSVP / Oddball paradigm	Surprise > Search trials	3T	*Event-related*	N
Asplund et al. (2010)	30	NA	RSVP / Oddball paradigm	Search trials > Baseline	3T	*Event-related*	N
Asplund et al. (2010)	6	NA	RSVP / Oddball paradigm	Search trials > Baseline	3T	*Event-related*	N
Asplund et al. (2010)	6	NA	RSVP / Oddball paradigm	Surprise > Search trials	3T	*Event-related*	N
Baldauf and Desimone (2014)	12	23-37	Object-based attention paradigm	Attend face/house blocks > Difficulty-mathced rare target detection	3T	Blocked	N
Bode and Haynes (2009)	12	26.4; 24-30	Task-switching paradigm	Cue presentation > Baseline	3T	*Event-related*	N
Bode and Haynes (2009)	12	26.4; 24-30	Task-switching paradigm	Target presentation > Baseline	3T	*Event-related*	N
Bode and Haynes (2009)	12	26.4; 24-30	Task-switching paradigm	Response > Baseline	3T	*Event-related*	N
Bollinger et al. (2010)	18	23.4 ± 3.06; 18-28	Working memory paradigm	FC with FFA: Stimulus known > Stimulus unknown	3T	*Event-related*	N
Bollinger et al. (2010)	18	23.4 ± 3.06; 18-28	Working memory paradigm	FC with FFA: Stimulus known > Passive view + stimulus unknown	3T	*Event-related*	N
Brass and Von Cramon (2002)	11	26.2 ± 3.06	Task-switching paradigm	Cue-only trials > Null events	3T	*Event-related*	N
Brass and Von Cramon (2002)	11	26.2 ± 3.06	Task-switching paradigm	Cue-target > Cue-only trials	3T	*Event-related*	N
Brass and Von Cramon (2002)	11	26.2 ± 3.06	Task-switching paradigm	Cue-target trials > Null events	3T	*Event-related*	N
Brass and Von Cramon (2002)	11	26.2 ± 3.06	Task-switching paradigm	Cue-target trials > No-cue-target trials	3T	*Event-related*	N
Brass and Von Cramon (2004)	14	24.4 ± 1.9	Task-switching paradigm	Meaning switch > Cue switch trials	3T	*Event-related*	N
Brass and Von Cramon (2004)	14	24.4 ± 1.9	Task-switching paradigm	Cue switch > Cue repetition trials	3T	*Event-related*	N
Brass and Von Cramon (2004)	14	24.4 ± 1.9	Task-switching paradigm	Switch > Repetition trials (single cue)	3T	*Event-related*	N
Chen et al. (2013)	23	21 ± 1.67	Stroop paradigm	Stimulus incongruent > Congruent trials (early stage)	3T	*Event-related*	N
Chen et al. (2013)	23	21 ± 1.67	Stroop paradigm	Response incongruent > Stimulus incongruent (early stage)	3T	*Event-related*	N
Chen et al. (2013)	23	21 ± 1.67	Stroop paradigm	Response incongruent > Stimulus incongruent (late stage)	3T	*Event-related*	N
Cole and Schneider (2007)	9	19-42	Working memory paradigm	Target switching > Non-switching trials (target non-occluded)	3T	Mixed blocked/event-related	N
Cole and Schneider (2007)	9	19-42	Working memory paradigm	Target switching > Non-switching trials (target occluded)	3T	Mixed blocked/event-related	N
Corradi-Dell'Acqua et al. (2015)	18	28; 18-52	Object-based attention paradigm	High saliency unattended > attended stimuli	3T	*Event-related*	N
Derrfuss and Von Cramon (2004)	19	20-36	Task-switching paradigm	Switch > Null event trials	3T	*Event-related*	N
Derrfuss and Von Cramon (2004)	19	20-36	Stroop paradigm	Incongruent > Neutral trials	3T	*Event-related*	N
Derrfuss and Von Cramon (2004)	19	20-36	Working memory paradigm	2-back > 0-back blocks	3T	Blocked	N
Derrfuss et al. (2012)	12	25.3 ± 2.4; 22-31	Stroop paradigm	Incongruent > Congruent trials	3T	*Event-related*	N
Han and Marois (2013)	14	20-32	RSVP paradigm	Discontinuous > Continuous & continuous-hard conditions	3T	*Event-related*	N
Han and Marois (2014)	14	20-32	RSVP / Oddball paradigm	Single-short oddball trials > Search no-target trials	3T	*Event-related*	N
Han and Marois (2014)	14	20-32	RSVP / Oddball paradigm	Target > Distractor trials	3T	*Event-related*	N
Han and Marois (2014)	6	19-35	RSVP / Oddball paradigm	Target > Distractor trials	3T	*Event-related*	N
Han et al. (2018)	20	22-33	RSVP / Oddball paradigm	Oddball > Search trials	3T	*Event-related*	N
Harding et al. (2016)	25	25.5 ± 4.4	Working memory paradigm	2-back > 0-back trials (main effect)	3T	*Event-related*	N
Henseler et al. (2011)	27	24.56 ± 2.53	Working memory paradigm	External attending to targets > Baseline	3T	Blocked	N
Henseler et al. (2011)	27	24.56 ± 2.53	Working memory paradigm	Internal orienting > Baseline	3T	Blocked	N
Henseler et al. (2011)	27	24.56 ± 2.53	Working memory paradigm	Internal > External attending (position task)	3T	Blocked	N
Kim et al. (2012)	16	23.6 ± 2.9; 18-35	Task-switching paradigm	Task switch > Non-switch trials (main effect)	3T	*Event-related*	N
Kim et al. (2012)	16	23.6 ± 2.9; 18-35	Stroop paradigm	Incongruent > Congruent trials (main effect)	3T	*Event-related*	N
Lin et al. (2019)	18	27.4 ± 6.6	Working memory paradigm	FC face cue and target > FC scene cue and target	3T	*Event-related*	Y
Melcher and Gruber (2006)	12	25.67 ± 1.88	Stroop paradigm	Incongruent > Congruent trials	3T	*Event-related*	N
Melcher and Gruber (2006)	12	25.67 ± 1.88	Oddball paradigm	Word-oddball vs. Oddball control	3T	*Event-related*	N
Melcher and Gruber (2006)	12	25.67 ± 1.88	Oddball paradigm	Color-oddball vs. Oddball control	3T	*Event-related*	N
Roth et al. (2006)	12	23; 19-34	Working memory paradigm	Sustanined working memory > Sustained feature detection	3T	Blocked	N
Roth et al. (2006)	12	23; 19-34	Working memory paradigm	Update > Cued maintenance	3T	*Event-related*	N
Sreenivasan et al. (2014)	16	22; 18-32	Working memory paradigm	Memory > Rotation discrimination trials	3T	Blocked	N
Stelzel et al. (2011)	48	F: 22 ± 1.99; M: 22.6 ± 1.99	Task-switching paradigm	Task switch > Repetition trials	3T	*Event-related*	N
Todd et al. (2011)	18	18-31	Working memory paradigm	Encoding period > Baseline	3T	*Event-related*	N
Wills et al. (2017)	22	18-30	Contingent capture paradigm	Salient target > Baseline	3T	*Event-related*	N
Yin et al. (2017)	26	21.3; 21-25	Task-switching paradigm	Task switch > Repetition trials	3T	*Event-related*	N
Zanto et al. (2010)	13	25; 20-31	Working memory paradigm	FC with V4: Attend > Ignore color	3T	*Event-related*	N
Zanto et al. (2010)	13	25; 20-31	Working memory paradigm	FC with V5: Attend > Ignore motion	3T	*Event-related*	N
Zanto et al. (2011)	20	24.25; 18-31	Working memory paradigm	FC with V4: Attend > Ignore color	3T	*Event-related*	N
Zanto et al. (2011)	20	24.25; 18-31	Working memory paradigm	FC with V5: Attend > Ignore motion	3T	*Event-related*	N
Zhang et al. (2018)	19	19-26	Feature-based attention paradigm	Stimulus block > Baseline	3T	Blocked	Y
Zhao et al. (2020)	13	21.54 ± 1.99	Working memory paradigm	High working memory load > Low working memory load	3T	*Event-related*	N

Supplementary Table 2 (Continued)

Study	Independent fMRI localizer	Field of View (FOV)	Results reported for	N° of foci	IFJ's activity lateralization	Software	Space
Armbruster et al. (2012)	N	192 mm	Whole-brain	15	Bilateral	SPM8	MNI
Armbruster et al. (2012)	N	192 mm	Whole-brain	9	Bilateral	SPM8	MNI
Armbruster et al. (2012)	N	192 mm	Whole-brain	13	Left	SPM8	MNI
Asplund et al. (2010)	N	240 mm	Whole-brain	17	Bilateral	BrainVoyager 4.9.1, QX 1.7.9	Talairach
Asplund et al. (2010)	N	240 mm	Whole-brain	8	Bilateral	BrainVoyager 4.9.1, QX 1.7.9	Talairach
Asplund et al. (2010)	N	240 mm	ROIs	8	Bilateral	BrainVoyager QX 1.11.4	Talairach
Asplund et al. (2010)	N	240 mm	ROIs	8	Bilateral	BrainVoyager QX 1.11.4	Talairach
Baldauf and Desimone (2014)	Y	N/A	IFJ only	2	Bilateral	SPM8	MNI
Bode and Haynes (2009)	N	N/A	Whole-brain	7	NA	SPM2	MNI
Bode and Haynes (2009)	N	N/A	Whole-brain	12	NA	SPM2	MNI
Bode and Haynes (2009)	N	N/A	Whole-brain	9	NA	SPM2	MNI
Bollinger et al. (2010)	N	230 mm	Whole-brain	17	Right	SPM5	MNI
Bollinger et al. (2010)	N	230 mm	Whole-brain	17	Right	SPM5	MNI
Brass and Von Cramon (2002)	N	192 mm	Whole-brain	22	Bilateral	LIPSIA	Talairach
Brass and Von Cramon (2002)	N	192 mm	Whole-brain	21	Left	LIPSIA	Talairach
Brass and Von Cramon (2002)	N	192 mm	Whole-brain	10	Bilateral	LIPSIA	Talairach
Brass and Von Cramon (2002)	N	192 mm	Whole-brain	9	Bilateral	LIPSIA	Talairach
Brass and Von Cramon (2004)	N	192 mm	ROIs	3	Left	LIPSIA	Talairach
Brass and Von Cramon (2004)	N	192 mm	ROIs	4	NA	LIPSIA	Talairach
Brass and Von Cramon (2004)	N	192 mm	ROIs	3	Left	LIPSIA	Talairach
Chen et al. (2013)	N	250 x 250 mm	Whole-brain	10	NA	SPM8	Talairach
Chen et al. (2013)	N	250 x 250 mm	Whole-brain	5	NA	SPM8	Talairach
Chen et al. (2013)	N	250 x 250 mm	Whole-brain	9	NA	SPM8	Talairach
Cole and Schneider (2007)	N	210 mm	Whole-brain	12	Bilateral	BrainVoyager QX	Talairach
Cole and Schneider (2007)	N	210 mm	Whole-brain	5	NA	BrainVoyager QX	Talairach
Corradi-Dell'Acqua et al. (2015)	N	N/A	Whole-brain	5	Bilateral	SPM8	MNI
Derrfuss and Von Cramon (2004)	N	192 mm	IFJ only	2	Bilateral	LIPSIA	Talairach
Derrfuss and Von Cramon (2004)	N	192 mm	IFJ only	2	Bilateral	LIPSIA	Talairach
Derrfuss and Von Cramon (2004)	N	192 mm	IFJ only	2	Bilateral	LIPSIA	Talairach
Derrfuss et al. (2012)	N	220 x 220 mm	IFJ only	1	Left	FSL	MNI
Han and Marois (2013)	Y	240 mm	Whole-brain	14	Bilateral	BrainVoyager QX 1.10	Talairach
Han and Marois (2014)	N	240 mm	Whole-brain	7	Bilateral	BrainVoyager QX 2.3	Talairach
Han and Marois (2014)	N	240 mm	Whole-brain	11	Bilateral	BrainVoyager QX 2.3	Talairach
Han and Marois (2014)	N	240 mm	Whole-brain	11	Bilateral	BrainVoyager QX 2.3	Talairach
Han et al. (2018)	N	240 mm	Whole-brain	6	Bilateral	FSL	MNI
Harding et al. (2016)	N	210 x 210 mm	Whole-brain	21	Bilateral	SPM8	MNI
Henseler et al. (2011)	N	192 mm	Whole-brain	7	NA	SPM2	MNI
Henseler et al. (2011)	N	192 mm	Whole-brain	8	NA	SPM2	MNI
Henseler et al. (2011)	N	192 mm	Whole-brain	15	Bilateral	SPM2	MNI
Kim et al. (2012)	N	224 mm	Whole-brain	20	Left	SPM5	MNI
Kim et al. (2012)	N	224 mm	Whole-brain	22	Bilateral	SPM5	MNI
Lin et al. (2019)	N	220 mm	Whole-brain	9	Right	FSL	MNI
Melcher and Gruber (2006)	N	192 mm	Whole-brain	13	NA	SPM2	MNI
Melcher and Gruber (2006)	N	192 mm	Whole-brain	19	Bilateral	SPM2	MNI
Melcher and Gruber (2006)	N	192 mm	Whole-brain	29	Bilateral	SPM2	MNI
Roth et al. (2006)	N	N/A	Whole-brain	15	Left	AFNI	Talairach
Roth et al. (2006)	N	N/A	Whole-brain	11	Left	AFNI	Talairach
Sreenivasan et al. (2014)	N	N/A	ROIs	13	Right	AFNI	Talairach
Stelzel et al. (2011)	N	240 mm	Whole-brain	5	Left	SPM5	MNI
Todd et al. (2011)	N	240 mm	Whole-brain	27	Bilateral	BrainVoyager QX v.1.09	Talairach
Wills et al. (2017)	N	220 mm	ROIs	13	Bilateral	AFNI	Talairach
Yin et al. (2017)	N	192 mm	Whole-brain	15	Left	SPM8	MNI
Zanto et al. (2010)	Y	230 mm	Whole-brain	5	Right	SPM5	MNI
Zanto et al. (2010)	Y	230 mm	Whole-brain	8	Bilateral	SPM5	MNI
Zanto et al. (2011)	Y	230 mm	Whole-brain	18	Right	SPM5	MNI
Zanto et al. (2011)	Y	230 mm	Whole-brain	16	Bilateral	SPM5	MNI
Zhang et al. (2018)	N	192 mm	ROIs	8	Bilateral	BrainVoyager QX	Talairach
Zhao et al. (2020)	N	210 mm	Whole-brain	7	Bilateral	SPM8	MNI

0. Mapping of the coordinates from Talairach to the MNI152 standard space

We converted all the Talairach coordinates to the MNI152 space using the Lancaster transform (Laird et al. 2010; Lancaster et al. 2007) as implemented by the function provided in the GingerALE software (v. 3.0.2; Eickhoff et al. 2012). We note that after the SPM2 version, the MNI templates distributed are consistent across FSL and SPM software packages, being compliant with the ICBM-152 coordinate space (Fonov et al. 2009), so for any later version of these packages we used the Talairach to MNI FSL transform. Where other software packages were used for spatial normalization, we again employed the Talairach to MNI FSL transform for consistency, as the other transformation provided in GingerALE represents a pooled FSL/SPM transformation (Lancaster et al. 2007) that would only lead to systematic displacement of the coordinates. In only two cases (i.e., Manoach et al. 2007; Mao et al. 2007) the studies employed the mapping from MNI to Talairach developed by Brett et al. (2002). These coordinates were therefore mapped back to the MNI space using this specific transformation, as recommended in the GingerALE user manual.

1. ALE contrast analyses method

Since we were interested in exploring potential dissociations in the prefrontal cortex, we carried out several ALE contrast analyses. For all these contrast analyses, we first computed separate ALE results for each function or contrast of interest by setting the ALE parameters to 10000 threshold permutations and a cluster-level FWE of 0.01 with $p < 0.001$. The experiments included in either analysis were randomly split into two groups of equal size to run a third pooled ALE analysis using the same parameters. The ALE scores of these two groups were then subtracted voxel-wise from each other 10000 times to create a null distribution of the difference between them. We used a threshold of $p < 0.01$ to infer significant differences between the two groups with a minimum cluster size of 25 mm^3 (as in Cieslik et al. 2016). The voxel-wise minimum of the ALE scores between the samples was used to create the conjunction image that indicates the similarity between the two groups of experiments.

2. ALE control and contrast analyses - FEF sample

1. **Cluster-level FWE control analysis.** In our main FEF localizer analysis, we applied voxel-level FWE correction, as this method allowed us a fine-grained assessment of the significant activations near the FEF. However, in the case of regions that are under-reported in the literature (i.e., the iFEF; Derrfuss et al. 2012), the use of a voxel-level FWE correction method may impose a too-conservative threshold that would prevent us from detecting clusters of activity that are also consistently activated in the FEF localizer sample. For instance, this could be due to the fact that most studies where an FEF functional localizer was employed tend to report only a pair of bilateral foci, leaving out other regions potentially active in the task (see Table S1 for the studies that have only reported part of their results), thus biasing the results in favor of the main activation cluster. In this first control analysis, we applied a less

stringent multiple comparison correction method in order to reveal other consistently active peaks in neighboring frontal sites, namely cluster-level FWE. This correction method offers an optimal balance between sensitivity and specificity in ALE analyses to detect regions that may be under-reported (Eickhoff et al. 2016). As we introduced above, since the iFEF is under-reported in the fMRI literature, we hypothesized that this control analysis could help uncover these activations. We therefore repeated the same ALE procedure on the FEF localizer sample setting the ALE parameters to a p-value of 0.001, with 10000 threshold permutations and applying a cluster-level FWE of 0.01.

2. **ALE contrast analysis - antisaccades vs prosaccades.** The second control analysis was intended as a replication of the ALE meta-analysis by Cieslik et al. (2016), who performed an ALE contrast analysis between experiments investigating prosaccades > fixation vs antisaccades > prosaccades in two equal samples of 12 experiments (see also Jamadar et al. 2013, for previous results). We note that, unlike Cieslik et al. (2016), we did not include PET studies, and also 1.5T fMRI studies to increase the spatial accuracy of our analysis (see Supplementary Table 4 below for the details of the experiments included).

3. **ALE contrast analysis - prosaccades vs covert spatial attention.** In our main localizer analysis, we did not include covert spatial attention tasks as these aren't considered the gold standard to localize the FEF. However, covert spatial attention paradigms are often employed to localize all the main nodes of the dorsal attention network, including the FEF (Corbetta and Shulman 2002). Therefore, studies using adaptations of the spatial cueing paradigm (Posner et al. 1980) were included in this analysis (see Supplementary Table 5). Running a contrast analysis between prosaccades and covert spatial attention paradigm allowed us to compare the topography and the sources of overt and covert attention near the putative FEF. For this analysis, we used prosaccades > fixation vs valid > neutral/invalid trials contrasts.

3. ALE contrast analyses - IFJ sample

Given the heterogeneity of the experiments included in the IFJ localizer sample, we carried out exploratory ALE contrast analyses by splitting up the IFJ sample according to the function investigated by each paradigm (i.e., oddball/attention vs working memory vs task-switching and Stroop paradigms; see Table S2) to see whether these paradigms elicited activity in distinct regions near the putative IFJ and to assess potential lateralization patterns. Task-switching and Stroop paradigms were grouped based on the results from Derrfuss et al. (2005), and we refer to this group of experiments as targeting cognitive control. In the first group of experiments (oddball/attention), most of the oddball experiments contrasted oddball > target trials and target trials > baseline (see for example Asplund et al. 2010), thus tapping on both stimulus-driven and goal-driven attentional mechanisms. There were also some examples of blocked design cueing paradigms contrasting target vs fixation blocks (Baldauf and Desimone 2014; Zhang et al. 2018). In the working memory group, the majority of

experiments contrasted the functional connectivity between perceptual seed regions (V4, V5, FFA) in the attended vs ignored conditions (see for example Bollinger et al. 2010; Lin et al. 2019; Zanto et al. 2010, 2011). We note that even though these studies were based on a second-level contrasts analysis, and thus involve some form of masking (i.e., the restriction to a specific brain region for the assessment of the significance level, hence spatial bias), the significant correlations with each seed were assessed over the whole-brain, making the localization of the IFJ with this method likely only slightly affected by this issue, if at all. Indeed, as previously suggested by Nee et al. (2013), their results match well those from traditional univariate analyses. The cognitive control group was mostly composed of experiments contrasting switch > repeat trials and incongruent > congruent contrasts in Stroop paradigms.

We therefore ran three separate ALE contrast analyses:

1. **Oddball/attention vs working memory** (11 vs 12 experiments):
2. **Working memory vs cognitive control** (12 vs 11 experiments), and;
3. **Oddball/attention vs cognitive control** (11 vs 11 experiments).

Supplementary Table 3 List of the experiments included in the prosaccade > fixation analysis

Study	N	Age	Paradigm	Contrast	Scanner	Design
Alkan et al. (2011)	8	26 ± 4	Functional localizer	Prosaccades > Fixation	3T	Blocked
Amiez and Petrides (2018)	13	22.6 ± 2.8	Functional localizer	Prosaccades > Fixation	3T	Blocked
Atmaca et al. (2013)	11	26.9; 22-33	Functional localizer	Prosaccades > Fixation	3T	Blocked
Bär et al. (2016)	14	22-56	Functional localizer	Prosaccades > Fixation	3T	Blocked
Berman et al. (1999)	11	25.6 ± 7.1; 18-43	Functional localizer	Prosaccades > Fixation	3T	Blocked
Braga et al. (2016)	20	26.2; 21-36	Functional localizer	Prosaccades > Fixation	3T	Blocked
Brown et al. (2006)	10	26; 22-33	Functional localizer	Prosaccades > Fixation	4T	*Event-related*
Connolly et al. (2000)	7	24.8 ± 3.2	Functional localizer	Prosaccades > Fixation	4T	Blocked
Connolly et al. (2002)	8	NA	Functional localizer	Prosaccades > Fixation	4T	Blocked
Connolly et al. (2005)	5	NA	Functional localizer	Prosaccades > Fixation	4T	Blocked
Connolly et al. (2007)	8	NA	Functional localizer	Prosaccades > Fixation	4T	Blocked
Christophel et al. (2018)	22	24.4 ± 0.83	Functional localizer	Prosaccades > Fixation	3T	*Event-related*
Duecker et al. (2013)	20	19-28	Functional localizer	Prosaccades > Fixation	3T	Blocked
Frannson et al. (2014)	24	26.7 ± 4.9	Functional localizer	Prosaccades > Fixation	3T	Blocked
Guo et al. (2012)	12	19-31	Functional localizer	Prosaccades > Fixation	3T	Blocked
Gurel et al. (2018)	16	23.2	Functional localizer	Prosaccades > Fixation	3T	Blocked
Heinen et al. (2006)	5	20-37	Functional localizer	Prosaccades > Fixation	3T	Blocked
Hubl et al. (2008)	7	31 ± 9	Functional localizer	Prosaccades > Fixation	3T	Blocked
Jamadar et al. (2015)	23	25.8; 18-43	Functional localizer	Prosaccades > Fixation	3T	*Event-related*
Jarvstad & Gilchrist (2019)	23	NA	Functional localizer	Prosaccades > Fixation	3T	Blocked
Kastner et al. (2007)	4	20-36	Functional localizer	Prosaccades > Fixation	3T	Blocked
Kurata et al. (2005)	6	29-38	Functional localizer	Prosaccades > Fixation	3T	Blocked
Levy et al. (2007)	4	24-43	Functional localizer	Prosaccades > Fixation	3T	Blocked
Matsuo et al. (2003)	12	20-42	Functional localizer	Prosaccades > Fixation	3T	Blocked
Pierce et al. (2019)	30	25.7 ± 4.1	Functional localizer	Prosaccades > Fixation	3T	Blocked
Schon et al. (2008)	17	21.29 ± 3.72	Functional localizer	Prosaccades > Fixation	3T	Blocked
Tamber-Rosenau et al. (2018)	8	28.5 ± 3.3	Functional localizer	Prosaccades > Fixation	3T	Blocked
Tark & Curtis (2009)	5	22-39	Functional localizer	Prosaccades > Fixation	3T	Blocked
Tibber et al. (2010)	16	21-40	Functional localizer	Prosaccades > Fixation	3T	Blocked

Supplementary Table 3 (Continued)

Study	Eye tracker	Results reported for	N° of foci	Multiple FEF foci	Software	Space
Alkan et al. (2011)	Y	Whole-brain	19	N	AFNI	Talairach
Amiez and Petrides (2018)	N	FEF foci	2	N	SPM12b	MNI
Atmaca et al. (2013)	N	Whole-brain	24	Y	SPM8	MNI
Bär et al. (2016)	N	Whole-brain	12	N	BrainVoyager QX 1.10	Talairach
Berman et al. (1999)	N	ROIs	14	Y	AFNI	Talairach
Braga et al. (2016)	Y	Whole-brain	7	N	FSL	MNI
Brown et al. (2006)	Y	Whole-brain	17	N	BrainVoyager 2000	Talairach
Connolly et al. (2000)	N	Whole-brain	7	Y	Stimulate	Talairach
Connolly et al. (2002)	N	ROIs	4	N	Stimulate	Talairach
Connolly et al. (2005)	Y	ROIs	5	N	Stimulate / Brain Voyager 4.9	Talairach
Connolly et al. (2007)	N	ROIs	2	Y	Stimulate / BrainVoyager 4.9 / QX	Talairach
Christophel et al. (2018)	N	FEF foci	2	N	SPM8	MNI
Duecker et al. (2013)	N	FEF foci	2	N	BrainVoyager QX 2.3	Talairach
Frannson et al. (2014)	Y	Whole-brain	8	N	SPM8	MNI
Guo et al. (2012)	Y	ROIs	6	N	SPM8	Talairach
Gurel et al. (2018)	Y	FEF foci	2	N	BrainVoyager QX	Talairach
Heinen et al. (2006)	Y	ROIs	4	N	VISTASOFT	Talairach
Hubl et al. (2008)	N	Whole-brain	9	N	BrainVoyager QX	Talairach
Jamadar et al. (2015)	Y	Whole-brain	47	N	SPM8	MNI
Jarvstad & Gilchrist (2019)	Y	Whole-brain	4	N	FSL v.5.06-1	MNI
Kastner et al. (2007)	Y	ROIs	5	N	AFNI	Talairach
Kurata et al. (2005)	N	Whole-brain	14	N	Advanced Visual Systems & AFNI	Talairach
Levy et al. (2007)	N	ROIs	5	N	BrainVoyager QX	Talairach
Matsuo et al. (2003)	N	FEF foci	1	N	SPM99	MNI
Pierce et al. (2019)	Y	Whole-brain	9	Y	SPM12	MNI
Schon et al. (2008)	N	Whole-brain	19	N	SPM2	MNI
Tamber-Rosenau et al. (2018)	Y	ROIs	6	N	BrainVoyager QX v.1.10.2–2.8	Talairach
Tark & Curtis (2009)	Y	FEF foci	2	N	Caret	MNI
Tibber et al. (2010)	Y	ROIs	9	N	SPM5	Talairach

Supplementary Table 4 List of the experiments included in the antisaccades > prosaccade analysis

Study	N	Age	Paradigm	Contrast	Scanner	Design
Brown et al. (2006)	10	26; 22-33	Antisaccade paradigm	Antisaccades > Prosaccades trials	4T	*Event-related*
Brown et al. (2007)	11	25; 20-28	Antisaccade paradigm	Antisaccades > Prosaccades trials	4T	*Event-related*
Brown et al. (2007)	11	25; 20-28	Antisaccade paradigm	Antisaccades > Prosaccades trials	4T	*Event-related*
Cameron et al. (2009)	11	22-30	Antisaccade paradigm	Antisaccades > Prosaccades trials	3T	*Event-related*
Ford et al. (2005)	10	28	Antisaccade paradigm	Antisaccades > Prosaccades trials	4T	*Event-related*
Manoach et al. (2007)	21	34.2 ± 12.6	Antisaccade paradigm	Antisaccades > Prosaccades trials	3T	*Event-related*
Neggers et al. (2012)	13	20-35	Antisaccade paradigm	Antisaccades > Prosaccades trials	3T	*Event-related*
Peirce & McDowell (2016)	35	19 ± 3.5	Antisaccade paradigm	Antisaccades > Prosaccades trials	3T	*Event-related*
Peirce & McDowell (2017)	35	19 ± 3.5	Antisaccade paradigm	Antisaccades > Prosaccades blocks	3T	Blocked
Salvia et al. (2020)	14	27.1 ± 2.7	Antisaccade paradigm	Antisaccades > Prosaccades trials	3T	*Event-related*

Supplementary Table 4 (Continued)

Study	Eye tracker	Results reported for	N° of foci	Software	Space
Brown et al. (2006)	Y	Whole-brain	15	BrainVoyager 2000	Talairach
Brown et al. (2007)	Y	Whole-brain	14	BrainVoyager 2000	Talairach
Brown et al. (2007)	Y	Whole-brain	11	BrainVoyager 2000	Talairach
Cameron et al. (2009)	Y	FEF foci	2	BrainVoyager 1.9	Talairach
Ford et al. (2005)	Y	Whole-brain	8	BrainVoyager 2000 v. 4.8	Talairach
Manoach et al. (2007)	Y	Whole-brain	7	FreeSurfer	Talairach
Neggers et al. (2012)	N	ROIs	13	SPM5	MNI
Peirce & McDowell (2016)	Y	Whole-brain	5	AFNI	Talairach
Peirce & McDowell (2017)	Y	Whole-brain	2	AFNI	Talairach
Salvia et al. (2020)	Y	Whole-brain	12	SPM12	MNI

Supplementary Table 5 List of the experiments included in the endogenous covert spatial attention analysis

Study	N	Age	Paradigm	Contrast	Scanner	Design
Chica et al. (2013)	18	25 ± 4	Spatial cueing	Cue > Jitter Fixation	3T	*Event-related*
Fan et al. (2005)	16	27.2 ± 5.7	Attentional network test	Spatial > Center cue	3T	*Event-related*
Ikkai & Curtis (2008)	14	21-35	Attentional cueing	Covert shift > Baseline	3T	*Event-related*
Mao et al. (2007)	12	24.3; 21-27	Spatial cueing	Attention to peripheral locations > Fixation	3T	Blocked
Mohanty et al. (2009)	13	27 ± 3.8	Spatial cueing / Visual search	Valid > Neutral trials	3T	*Event-related*
Vossel et al. (2012)	24	26.83; 20-37	Spatial cueing	Valid trials > Baseline	3T	*Event-related*
Wen et al. (2012)	12	20-28	Spatial cueing	Attend > Passive viewing blocks	3T	Blocked
Xuan et al. (2016)	24	26.3; 18-49	Attentional network test	Valid > Invalid trials	3T	*Event-related*

Supplementary Table 5 (Continued)

Study	Eye tracker	Results reported for	N° of foci	Multiple FEF foci	Software	Space
Chica et al. (2013)	N	Whole-brain	22	Y	SPM5	MNI
Fan et al. (2005)	N	Whole-brain	8	N	SPM99	MNI
Ikkai & Curtis (2008)	Y	ROIs	11	Y	Caret	MNI
Mao et al. (2007)	N	Whole-brain	6	N	SPM99	Talairach
Mohanty et al. (2009)	Y	Whole-brain	10	N	SPM5	MNI
Vossel et al. (2012)	Y	Whole-brain	6	N	SPM8	MNI
Wen et al. (2012)	N	ROIs	7	N	SPM2	MNI
Xuan et al. (2016)	N	Whole-brain	20	N	SPM8	MNI

Supplementary Table 6 FEF sample no ROI control analysis ALE results

Cluster	Macroanatomical location	Hemi	MNI152 coordinates			ALE value	Volume (mm³)	BA
			x	y	z			
1	Medial Frontal Gyrus	L/R	−2	0	58	0.0386	1360	6
2	Precentral Gyrus	L	−28	−6	56	0.0341	992	6
3	Middle Frontal Gyrus	R	28	−6	50	0.0308	960	6
	Precentral Gyrus	R	40	−4	50	0.0275		6
4	Precuneus	L	−22	−58	56	0.0306	488	7
5	Precuneus	R	26	−60	56	0.0223	112	7

Supplementary Table 7 IFJ sample no ROI control analysis ALE results

Cluster	Macroanatomical location	Hemi	MNI152 coordinates			ALE value	Volume (mm³)	BA
			x	y	z			
1	Precentral Gyrus	L	−42	6	30	0.0532	2632	6
2	Inferior Frontal Gyrus	R	46	10	26	0.0402	1672	9
	Precentral Gyrus	R	42	8	30	0.0389		6
3	Medial Frontal Gyrus	L	−2	18	46	0.0462	1656	6
4	Precentral Gyrus	L	−28	−4	54	0.0474	816	6
5	Claustrum	R	32	22	−4	0.0348	456	*
6	Precuneus	L	−26	−66	44	0.0302	456	7
7	Inferior Parietal Lobule	L	−32	−54	48	0.0348	384	7
8	Superior Parietal Lobule	R	34	−54	46	0.0323	312	7
9	Claustrum	L	−30	18	2	0.0317	264	*

4. ALE control and contrast analyses - FEF sample results

By repeating the ALE analysis using cluster-level FWE instead of voxel-level FWE, we were able to uncover bilateral activations ventral to the main FEF ALE peaks. These activations were extending from the sPCS to the posterior bank of the iPCS and were primarily localized in the iPCS (see Figure S3), corresponding to the iFEF (Kastner et al. 2007). This highlights the fact that, although these clusters may be under-reported in the literature (Derrfuss et al. 2012), they were nevertheless consistently activated in our sample of experiments.

Our two ALE contrast analyses show evidence of important spatial segregation and overlap within FEF for antisaccades, prosaccades and covert spatial attention contrasts (see Figures S4 and S5). Starting with the first contrast analysis, we would like to comment separately on the ALE results for antisaccades > prosaccades and the 'pure' prosaccade > fixation results, as these give important clues on how to interpret the ALE peaks from our

main analysis results (where we also included ‘mixed’ antisaccades & prosaccades > fixation and antisaccades > fixation contrasts to increase our sample size). In the prosaccades > fixation ALE results the FEF peaks were localized in: LH 1st ALE peak (−28, −6, 54); LH 2nd ALE peak (−52, 0, 40); RH 1st ALE peak (36, −4, 52); RH 2nd ALE peak (30, −4, 50); RH 3rd ALE peak (34, −2, 50). While the left hemisphere peaks match the main analysis results, in the right hemisphere the strongest convergence is now found more lateral compared to the main result ALE peak (30, −6, 50) suggesting a higher variability along this axis (see Figure S4, Panel A). In the antisaccades > prosaccades ALE results there were only two peaks localized within FEF, specifically in LH (−26, −2, 56), and RH (−28, −2, 54). These peaks were localized more anteriorly and medially compared to our main results (Figure S4, Panel B). This already suggests that there may be important dissociations between the activations derived from these contrasts. Indeed, when we directly contrasted these two samples, we found that the antisaccades > prosaccades and prosaccades > fixation experiments consistently activated segregated prefrontal clusters, as previously reported by other studies (Cieslik et al. 2016; Jamadar et al. 2013). While in the right hemisphere, the antisaccade cluster was medial relative to the prosaccade cluster, and largely overlapping at the junction of the sPCS with the SFS, this organization was less evident in the left hemisphere, where the two clusters overlapped near the same anatomical location, but with a segregated cluster for antisaccades localized in its anterior-medial part (see Figure S4, Panel C). Overall, these results suggest that the additional processes required for the antisaccade task (Munoz and Everling 2004), namely response inhibition and the execution of a saccade towards the opposite location relative to the target, consistently recruit the medial part of the FEF (McDowell et al. 2008). These processes may be mediated by distinct neural populations within the FEF (see Lowe and Schall 2018, for a classification in the macaque). We also found two bilateral segregated clusters in the medial prefrontal cortex for prosaccades localized in the posterior SCEF. The antisaccades clusters were instead localized in the right anterior SCEF. We did not find clusters in the posterior parietal cortex as in Cieslik et al. (2016), except for a cluster in the left precuneus/SPL, possibly due to the fact that our antisaccade sample was lower compared to that study (8 vs 12 experiments), and the influence of studies only reporting ROI analyses.

Our second ALE contrast analysis, namely between prosaccades and covert spatial attention activations (Figure S5, Panel C) reveals a clear pattern of overlap near the junction of the sPCS and SFS. These results are consistent with the hypothesis that covert and overt attention have a spatially common source within the FEF (Astafiev et al. 2003; Corbetta et al. 1998; de Haan et al. 2008). We also found evidence of segregated clusters, with covert attention clusters being mostly localized in the posterior bank of the sPCS in the right and to a smaller extent in the left hemispheres (not visible in Figure S5). These results strongly suggest that covert spatial attention paradigms may be equally adept as FEF functional localizers compared to the current prosaccade gold standard, although more trials may be needed to reliably elicit activations in all subjects due to the weaker nature of the signal measured (Beauchamp et al. 2001; De Haan et al. 2008).

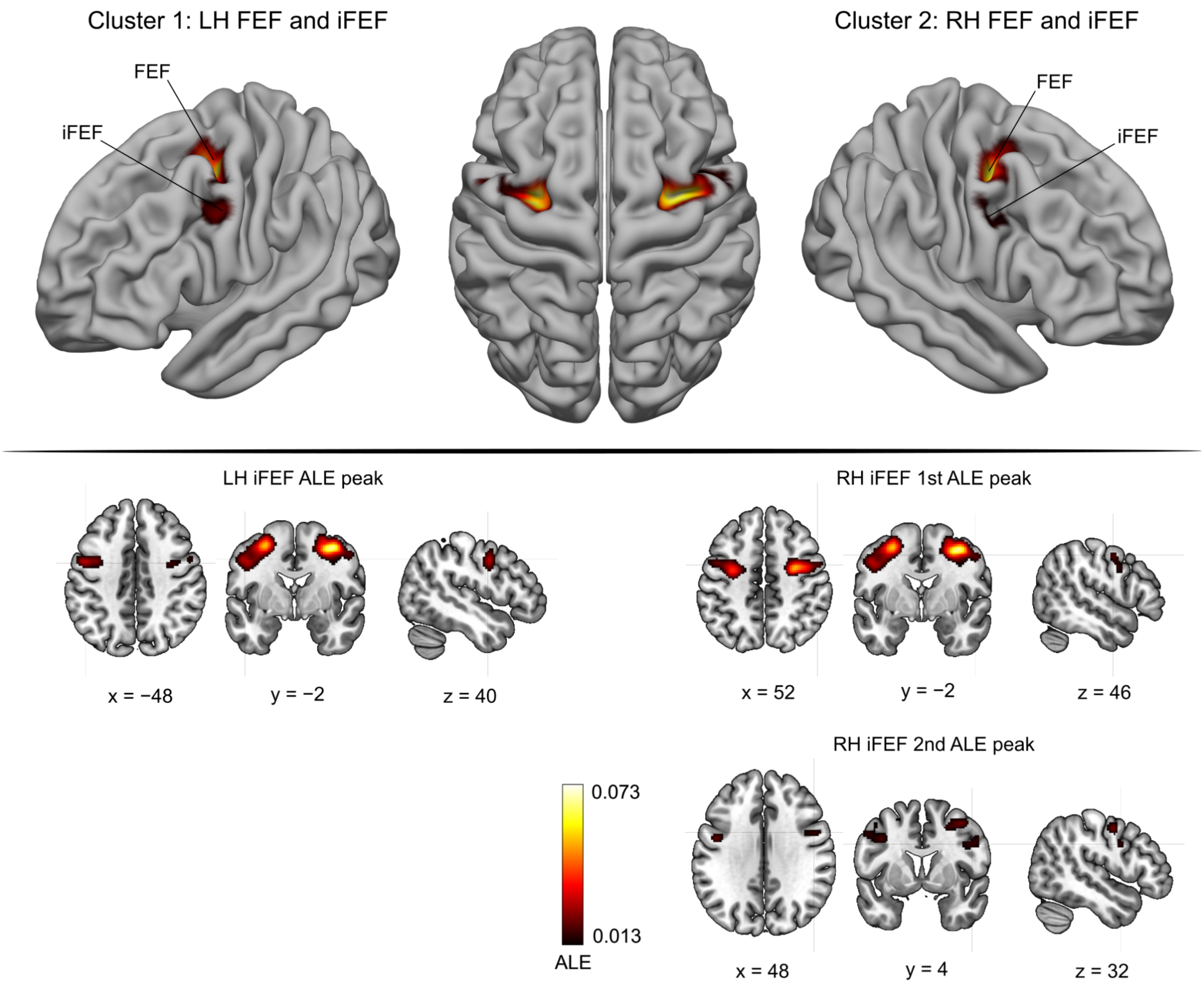

FIGURE S3 Results of the first FEF sample control analysis - main clusters of activation. Applying cluster-level FWE in the ALE analysis allowed us to uncover bilateral activations ventral to the main FEF peaks. These activations were extending from the sPCS to the posterior bank of the iPCS, and were primarily localized in the precentral gyrus. These results reveal the presence of consistent iFEF activations in the FEF localizer sample and three iFEF peaks (shown in the volumetric views of the figure)

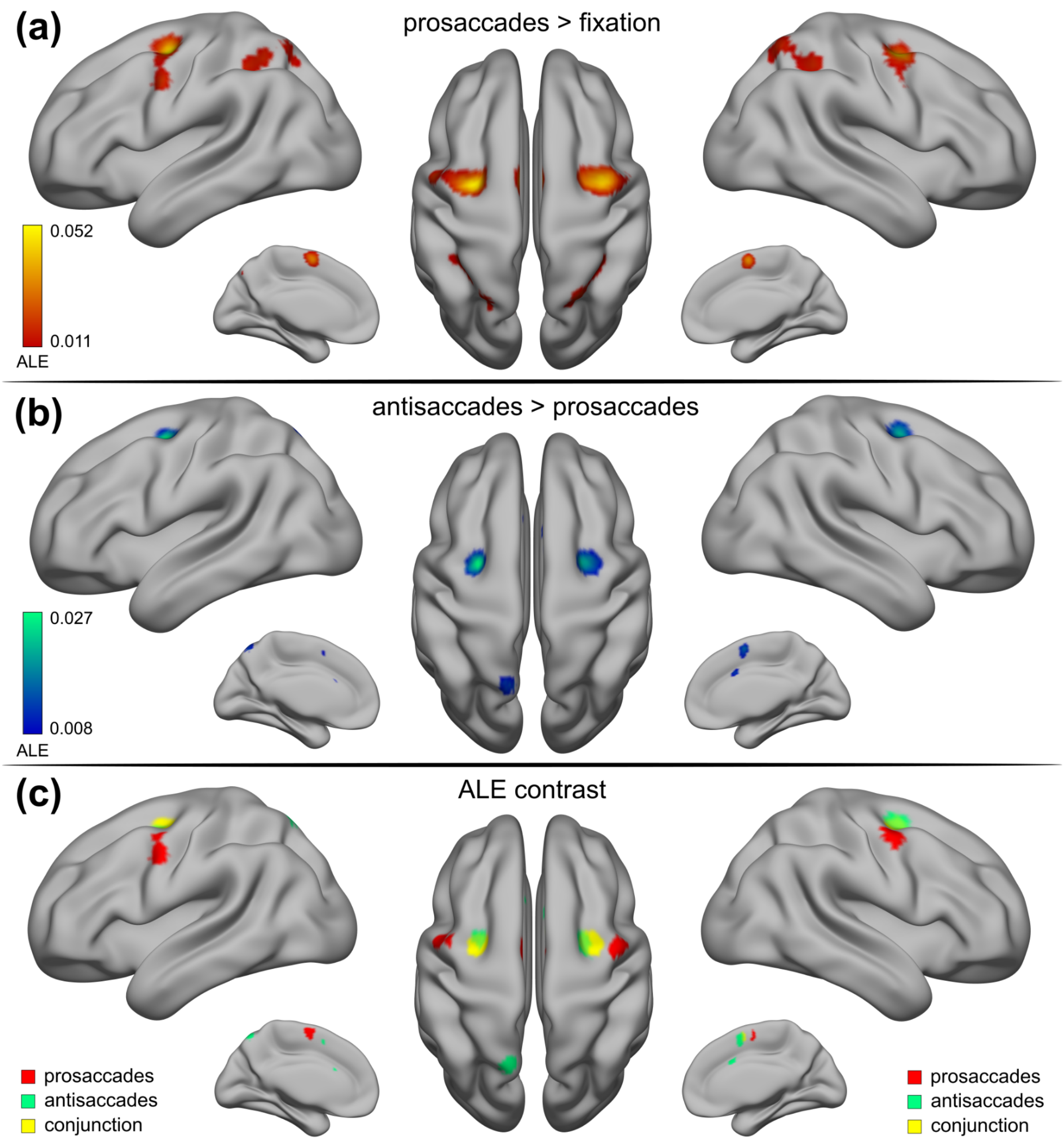

FIGURE S4 FEF sample contrast analysis between antisaccades > prosaccades vs prosaccades > fixation contrasts. **C** The clusters overlap in the bilateral posterior aspect of the SFS and sPCS. The contrast of these clusters showed that the antisaccades-related clusters were generally more medial and localized anterior to the prosaccades-related clusters. These results are consistent with those reported by Cieslik et al. (2016) and suggest that the additional mechanisms required for the antisaccade task are mediated by separate neural populations within the medial aspect of the FEF

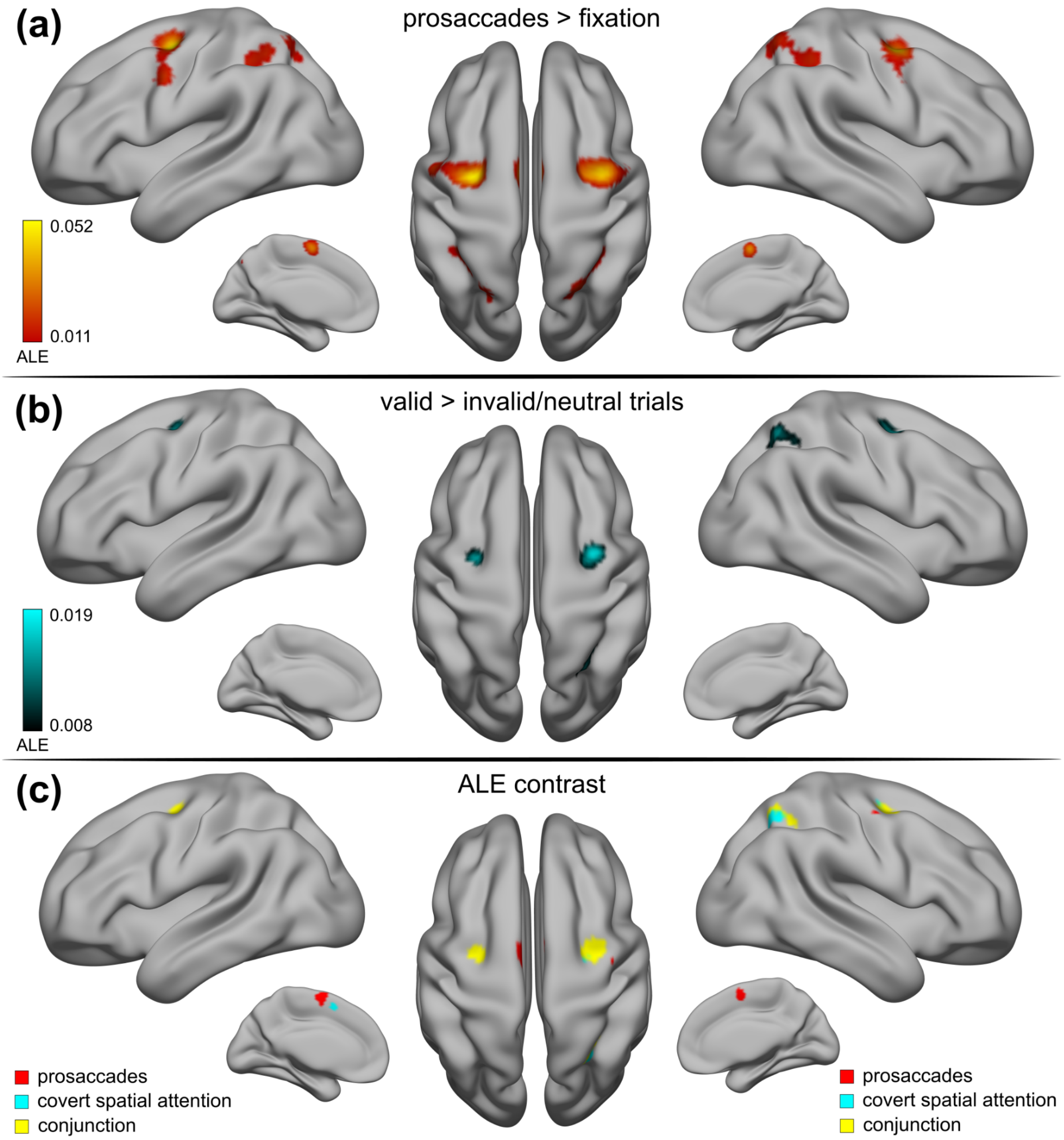

FIGURE S5 FEF sample contrast analysis between prosaccades > fixation vs valid > neutral/invalid trials in covert spatial attention task. **C** The clear overlap we found at the junction of the SFS and sPCS supports the hypothesis that covert and overt attention have a spatially common source within the FEF (Astafiev et al. 2003; Corbetta et al. 1998; de Haan et al. 2008)

5. ALE contrast analyses - IFJ sample results

By splitting up our IFJ localizer sample based on the paradigm employed (i.e., oddball/attention vs working memory vs cognitive control), we found some dissociations within the putative IFJ, as well as interesting lateralization patterns (see Figure S6). While oddball/attention paradigms gave rise to bilateral activations near the putative IFJ, working memory paradigms consistently activated only a right hemisphere cluster, whereas in contrast cognitive control paradigms were only consistently activating on a cluster in the left hemisphere. Remarkably, each of these clusters had a quite distinct spatial topography. In the left hemisphere, the cluster related to cognitive control (task-switching and Stroop paradigms) extended from the posterior bank of the superior iPCS to the junction of the iPCS with the IFS, where it overlapped with the oddball/attention cluster (see Figure S6, Panel A). This cluster further extended anteriorly and ventrally. The same arrangement was approximately found in the right hemisphere, in which a posterior-dorsal working memory limited cluster overlapped with the oddball/attention cluster just above the junction of the iPCS with the IFS (see Figure S6, Panel B). The oddball/attention cluster again extended anteriorly and ventrally. Finally, the contrast between working memory and cognitive control only revealed overlap in the left paracentral and midcingulate cortex, as the other clusters were localized within the right and left hemispheres, respectively (see Figure S6, Panel C).

Based on the results from these ALE contrast analyses and their comparisons with our main results, we would like to offer some suggestions on how to effectively localize the IFJ and segregate it from adjacent brain regions. First of all, at the general level, contrasts involving two demanding experimental conditions (e.g., task switch > repeat trials) may be more appropriate to measure activity within the IFJ compared to experimental trials vs passive fixation conditions. Secondly, an even more stringent way to isolate this region would be to compare experimental conditions that are matched in difficulty, thus avoiding contamination from non-specific cognitive load effects (as in Baldauf and Desimone 2014). Oddball/attention paradigms seemed to tap on the same IFJ cluster as found in our main localizer (see Figure S6, Panel A). However, in the experiments we analyzed, these tasks usually contrast activity between oddball and target trials, leading to a low number of trials that are used as functional localizers within each run as a result (Han and Marois 2014). An additional problem may be caused by spatial smoothing, which would lead to merging activity with the IFG, a node classically viewed as belonging to the ventral attention network (Corbetta and Shulman 2002), which is also significantly activated in these paradigms (Levy and Wagner 2011). Therefore, a more straightforward way to isolate the IFJ from the IFG may be achieved by administering top-down feature- and object-based attention tasks (Baldauf and Desimone 2014; Liu et al. 2011; Liu 2016; Zhang et al. 2018) and contrasting valid > invalid trials collapsed across the stimuli features/dimensions (e.g., similarly to the approach reported in Zanto et al. 2010). Posteriorly, another important issue is how to segregate IFJ activity from the iFEF (or the PEF in the MMP1 taxonomy). Derrfuss et al. (2012) reported that by employing a Stroop paradigm and contrasting incongruent > congruent trials, they were able to isolate activity from the adjacent iFEF (which was activated by the execution of voluntary saccades in darkness) in the native space in all the

subjects they analyzed. These results show a reliable way to infer the posterior IFJ functional border. As suggested previously however in the case of the FEF results, the presence of significant voxels within the iFEF is particularly difficult to interpret given that, in the IFJ localizer sample, only two out of 32 experiments (see Table S2) employed strict monitoring of eye movements in the scanner. The possibility that the convergence in iFEF in this sample may be due to this confound cannot be therefore completely ruled out (Amiez and Petrides 2009; Kato and Miyauchi 2003). Therefore we suggest that future localization approaches aimed at separating IFJ and iFEF activations must ensure a robust way to prevent data contamination from oculomotor artifacts. Finally, as suggested in our Discussion, the combination of different tasks and the manipulation of task difficulty seems a promising approach to delineate the IFJ based on a conjunction analysis (as in Stiers and Goulas 2018).

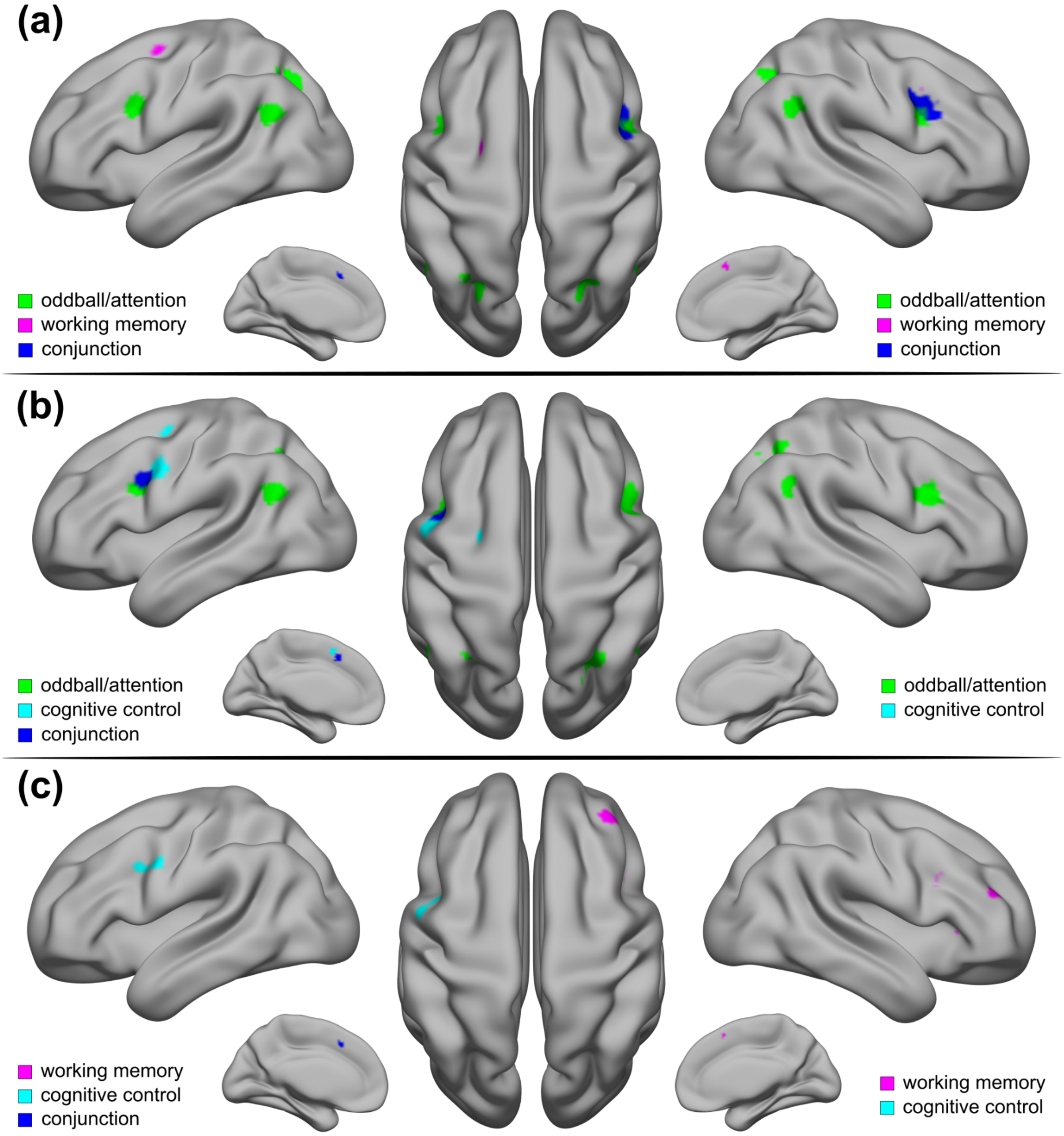

FIGURE S6 Results of the IFJ sample control analysis. The IFJ sample was split into three groups of experiments based on the paradigm employed (oddball/attention, working memory and cognitive control) to explore potential dissociations between them near the IFJ. In line with this possibility, we found a lateralized cluster involved in cognitive control (i.e., task-switching/Stroop paradigms) in the left hemisphere, and a cluster involved in working memory (mainly n-back contrasts) in the right hemisphere. These clusters overlapped with a bilateral cluster associated with oddball/attention experiments at the iPCS and IFS junction, including the IFJ. The oddball/attention clusters were however generally anterior and ventral relative to their location in both hemispheres (see **A** and **B**)

6. MACM contrast analysis results

To quantitatively examine the dissociations in the MACM patterns of FEF and IFJ for each hemisphere, we also ran an ALE contrast analysis between the LH FEF vs LH IFJ, and RH FEF vs RH IFJ coactivation patterns. Our results generally confirm what can be seen in Figure 5. The LH IFJ coactivated with the RH IFJ and the LH FEF, whereas the LH FEF coactivated with the RH FEF, and LH and RH IFJ. The LH IFJ had also additional coactivations with the insular cortex. Medially, there was a posterior cluster in the SCEF differentially coactivated with the LH FEF, and two clusters in the anterior and mid cingulate cortex coactivated with the LH IFJ. Posteriorly, we found common activations in the IPL, but crucially, dissociations in the precuneus/SPL (for the LH FEF) and the fusiform face complex, areas TE2p and V8, and the parahippocampal areas (for the LH IFJ). We found similar results in the right hemisphere. The RH FEF coactivated with the LH FEF, and the LH and RH IFJ. The RH IFJ only coactivated with the LH IFJ and had additional coactivations with the insular cortex. There were differential coactivations of the RH FEF with the precuneus/SPL and the lateral intraparietal area and common coactivations in the left SPL.

7. MACM decoding results

Performing a reverse inference on the coactivation patterns of the LH FEF showed that the prevalent association was in the ‘action’ behavioral domain (see Figure 5, right side of panel A), namely execution.unspecified. In the ‘cognition’ domain, there were four prominent associations with attention, working memory, reasoning and spatial cognition. Finally, in the ‘perception’ domain, the two highest associations were with vision.motion and vision.shape. The behavioral domain associations with the coactivation patterns of the RH FEF (see Figure 5, right side of panel B) were very similar to the LH FEF. Again, the primary association in the ‘action’ domain was with execution.unspecified. The prevalent association was however in the ‘cognition’ domain with attention, followed by working memory, language.speech and by reasoning. As for the previous seed, the two highest associations were with vision.motion and vision.shape in the ‘perception’ domain.

The functional decoding of the LH and RH IFJ coactivation patterns uncovered associations with similar behavioral domains, although with some interesting differences in their predominance. The LH IFJ coactivations had the highest association with attention in the ‘cognition’ domain (see Figure 5, right side of panel C), followed by language.semantics, working memory, and language.speech. The next strongest association was in the ‘emotion’ domain with positive.reward/gain. Then, there were significant associations in the ‘perception’ domain with vision.shape and vision.unspecified and audition. Finally, in the ‘action’ domain we found the most prevalent associations with inhibition and execution.unspecified. As for the previous seed, the RH IFJ had the highest association with attention in the ‘cognition’ domain (see Figure 5, right side of panel D). In the same domain, there were also strong associations with working memory, language.semantics, reasoning, and language.speech. Next, we found two prominent associations in the ‘action’ domain with inhibition and execution.unspecified. Again, we found an association with

positive.reward/gain in the ‘emotion’ domain. Lastly, the strongest associations in the ‘perception’ domain were with vision.shape, audition and somesthesis.pain.

8. Limitations

While we believe that the present study significantly advances our understanding of the localization and spatial organization of areas in the posterior-lateral PFC, we would like to acknowledge its limitations. First of all, even though the inclusion of ROI analyses is not recommended according to the best practices reported in Muller et al. (2018), we nevertheless decided to include them to increase our sample size, as we were mainly interested in inferring the localization of their peaks. When we excluded them in a control analysis (reported in the section 4 above), we showed that the inferred ALE remained virtually identical. We note however that the inclusion of these studies may have inflated the ALE values associated with the FEF and IFJ clusters in our main results, and potentially also their spatial extent. A second limitation of our study is that although we strived to include as many functional localizers as possible, particularly in the IFJ sample many of the included coordinates were only based on the results of the main fMRI task, and not an independent fMRI localizer. It is not clear to what extent these IFJ activations can be replicated, and there may be a file drawer problem in this sample, which is typically an issue in ALE meta-analyses (Acar et al. 2018). Finally, even though we tried to include experiments that tapped onto similar cognitive functions (i.e., spatial attention and oculomotor control for the FEF, and attention, working memory and cognitive control for the IFJ) to infer spatial convergence within FEF and IFJ, these experiments didn’t measure a single cognitive function but rather a collection of several distinct functions and sub-processes, some of which share an important degree of overlap. The detailed investigation of each separate component of these functions/processes, which are often subsumed under the umbrella term of executive functions, would require examining a broader number of paradigms than we included in our analyses, and could lead to further insights into anatomo-functional dissociations in the lateral PFC.

References

Acar F, Seurinck R, Eickhoff SB, Moerkerke B (2018) Assessing robustness against potential publication bias in Activation Likelihood Estimation (ALE) meta-analyses for fMRI. PLoS One 13:1–23. https://doi.org/10.1371/journal.pone.0208177

Alkan Y, Biswal BB, Alvarez TL (2011) Differentiation between vergence and saccadic functional activity within the human frontal eye fields and midbrain revealed through fMRI. PLoS One 6:1-14. https://doi.org/10.1371/journal.pone.002586

Amiez C, Petrides M (2018) Functional rostro-caudal gradient in the human posterior lateral frontal cortex. Brain Struct Funct 223:1487–1499. https://doi.org/10.1007/s00429-017-1567-z

Armbruster DJN, Ueltzhöffer K, Basten U, Fiebach CJ (2012) Prefrontal cortical mechanisms underlying individual differences in cognitive flexibility and stability. J Cogn Neurosci 24:2385–2399. https://doi.org/10.1162/jocn_a_00286

Asplund CL, Todd JJ, Snyder AP, Marois R (2010) A central role for the lateral prefrontal cortex in goal-directed and stimulus-driven attention. Nat Neurosci 13:507–512. https://doi.org/10.1038/nn.2509

Atmaca S, Stadler W, Keitel A, et al (2013) Prediction processes during multiple object tracking (MOT): Involvement of dorsal and ventral premotor cortices. Brain Behav 3:683–700. https://doi.org/10.1002/brb3.180

Bär S, Hauf M, Barton JJS, Abegg M (2016) The neural network of saccadic foreknowledge. Exp Brain Res 234:409–418. https://doi.org/10.1007/s00221-015-4468-5

Berman RA, Colby CL, Genovese CR, et al (1999) Cortical networks subserving pursuit and saccadic eye movements in humans: An FMRI study. Hum Brain Mapp 8:209–225. https://doi.org/10.1002/(SICI)1097-0193(1999)8:4<209::AID-HBM5>3.0.CO;2-0

Bode S, Haynes JD (2009) Decoding sequential stages of task preparation in the human brain. Neuroimage 45:606–613. https://doi.org/10.1016/j.neuroimage.2008.11.031

Bollinger J, Rubens MT, Zanto TP, Gazzaley A (2010) Expectation-driven changes in cortical functional connectivity influence working memory and long-term memory performance. J Neurosci 30:14399–14410. https://doi.org/10.1523/JNEUROSCI.1547-10.2010

Braga RM, Fu RZ, Seemungal BM, et al (2016) Eye movements during auditory attention predict individual differences in dorsal attention network activity. Front Hum Neurosci 10:1–13. https://doi.org/10.3389/fnhum.2016.00164

Brass M, Von Cramon DY (2002) The role of the frontal cortex in task preparation. Cereb Cortex 12:908–914. https://doi.org/10.1093/cercor/12.9.908

Brass M, Von Cramon DY (2004) Decomposing components of task preparation with functional magnetic resonance imaging. J Cogn Neurosci 16:609–620. https://doi.org/10.1162/089892904323057335

Brett M, Johnsrude IS, Owen AM (2002) The problem of functional localization in the human brain. Nat Rev Neurosci 3:243–249. https://doi.org/10.1038/nrn756

Brown MRG, Goltz HC, Vilis T, et al (2006) Inhibition and generation of saccades: Rapid event-related fMRI of prosaccades, antisaccades, and nogo trials. Neuroimage 33:644–659. https://doi.org/10.1016/j.neuroimage.2006.07.002

Brown MRG, Vilis T, Everling S (2007) Frontoparietal activation with preparation for antisaccades. J Neurophysiol 98:1751–1762. https://doi.org/10.1152/jn.00460.2007

Cameron IGM, Coe BC, Watanabe M, et al (2009) Role of the basal ganglia in switching a planned response. Eur J Neurosci 29:2413–2425. https://doi.org/10.1111/j.1460-9568.2009.06776.x

Chen Z, Lei X, Ding C, et al (2013) The neural mechanisms of semantic and response conflicts: An fMRI study of practice-related effects in the Stroop task. Neuroimage 66:577–584. https://doi.org/10.1016/j.neuroimage.2012.10.028

Chica AB, Paz-Alonso PM, Valero-Cabré A, Bartolomeo P (2013) Neural bases of the interactions between spatial attention and conscious perception. Cereb Cortex 23:1269–1279. https://doi.org/10.1093/cercor/bhs087

Connolly JD, Goodale MA, Desouza JFX, et al (2000) A comparison of frontoparietal fMRI activation during anti-saccades and anti-pointing. J Neurophysiol 84:1645–1655. https://doi.org/10.1152/jn.2000.84.3.1645

Connolly JD, Goodale MA, Menon RS, Munoz DP (2002) Human fMRI evidence for the neural correlates of preparatory set. Nat Neurosci 5:1345–1352. https://doi.org/10.1038/nn969

Connolly JD, Goodale MA, Goltz HC, Munoz DP (2005) fMRI activation in the human frontal eye field is correlated with saccadic reaction time. J Neurophysiol 94:605–611. https://doi.org/10.1152/jn.00830.2004

Connolly JD, Goodale MA, Cant JS, Munoz DP (2007) Effector-specific fields for motor preparation in the human frontal cortex. Neuroimage 34:1209–1219. https://doi.org/10.1016/j.neuroimage.2006.10.001

Corradi-Dell'Acqua C, Fink GR, Weidner R (2015) Selecting category specific visual information: Top-down and bottom-up control of object based attention. Conscious Cogn 35:330–341. https://doi.org/10.1016/j.concog.2015.02.006

Christophel TB, Allefeld C, Endisch C, Haynes JD (2018) View-Independent Working Memory Representations of Artificial Shapes in Prefrontal and Posterior Regions of the Human Brain. Cereb Cortex 28:2146–2161. https://doi.org/10.1093/cercor/bhx119

Curtis CE, Connolly JD (2008) Saccade preparation signals in the human frontal and parietal cortices. J Neurophysiol 99:133–145. https://doi.org/10.1152/jn.00899.2007

Derrfuss J, Brass M, Yves Von Cramon D (2004) Cognitive control in the posterior frontolateral cortex: Evidence from common activations in task coordination, interference control, and working memory. Neuroimage 23:604–612. https://doi.org/10.1016/j.neuroimage.2004.06.007

Derrfuss J, Vogt VL, Fiebach CJ, et al (2012) Functional organization of the left inferior precentral sulcus: Dissociating the inferior frontal eye field and the inferior frontal junction. Neuroimage 59:3829–3837. https://doi.org/10.1016/j.neuroimage.2011.11.051

DeSouza, JF, Menon, RS, Everling, S (2003). Preparatory set associated with pro-saccades and anti-saccades in humans investigated with event-related FMRI. J Neurophysiol 89:1016-1023. https://doi.org/10.1152/jn.00562.2002

Duecker F, Formisano E, Sack AT (2013) Hemispheric differences in the voluntary control of spatial attention: Direct evidence for a right-hemispheric dominance within frontal cortex. J Cogn Neurosci 25:1332–1342. https://doi.org/10.1162/jocn_a_00402

Fan J, Mccandliss TBD, Fossella J, et al (2005) The activation of attentional networks. Neuroimage 26:471–479. https://doi.org/10.1016/j.neuroimage.2005.02.004

Fernandez-Ruiz J, Peltsch A, Alahyane N, et al (2018) Age related prefrontal compensatory mechanisms for inhibitory control in the antisaccade task. Neuroimage 165:92–101. https://doi.org/10.1016/j.neuroimage.2017.10.001

Fonov V, Evans A, McKinstry R, et al (2009) Unbiased nonlinear average age-appropriate brain templates from birth to adulthood. Neuroimage 47:S102. https://doi.org/10.1016/s1053-8119(09)70884-5

Fransson P, Flodin P, Seimyr GÖ, Pansell T (2014) Slow fluctuations in eye position and resting-state functional magnetic resonance imaging brain activity during visual fixation. Eur J Neurosci 40:3828–3835. https://doi.org/10.1111/ejn.12745

Ford KA, Goltz HC, Brown MRG, Everling S (2005) Neural processes associated with antisaccade task performance investigated with event-related fMRI. J Neurophysiol 94:429–440. https://doi.org/10.1152/jn.00471.2004

Furlan M, Smith AT, Walker R (2016) An fMRI investigation of preparatory set in the human cerebral cortex and superior colliculus for pro- and anti-saccades. PLoS One 11:1–25. https://doi.org/10.1371/journal.pone.0158337

Guo F, Preston TJ, Das K, et al (2012) Feature-Independent Neural Coding of Target Detection during Search of Natural Scenes. J Neurosci 32:9499–9510. https://doi.org/10.1523/JNEUROSCI.5876-11.2012

Gurel SC, Castelo-Branco M, Sack AT, Duecker F (2018) Assessing the Functional Role of Frontal Eye Fields in Voluntary and Reflexive Saccades Using Continuous Theta Burst Stimulation. Front Neurosci 12:1–11. https://doi.org/10.3389/fnins.2018.00944

Hakvoort Schwerdtfeger RM, Alahyane N, Brien DC, et al (2013) Preparatory neural networks are impaired in adults with attention-deficit/hyperactivity disorder during the antisaccade task. NeuroImage Clin 2:63–78. https://doi.org/10.1016/j.nicl.2012.10.006

Han SW, Marois R (2013) Dissociation between process-based and data-based limitations for conscious perception in the human brain. Neuroimage 64:399–406. https://doi.org/10.1016/j.neuroimage.2012.09.016

Han SW, Marois R (2014) Functional fractionation of the stimulus-driven attention network. J Neurosci 34:6958–6969. https://doi.org/10.1523/JNEUROSCI.4975-13.2014

Han SW, Shin H, Jeong D, et al (2018) Neural substrates of purely endogenous, self-regulatory control of attention. Sci Rep 8:1–10. https://doi.org/10.1038/s41598-018-19508-6

Harding IH, Harrison BJ, Breakspear M, et al (2016) Cortical Representations of Cognitive Control and Working Memory Are Dependent Yet Non-Interacting. Cereb Cortex 26:557–565. https://doi.org/10.1093/cercor/bhu208

Heinen SJ, Rowland J, Lee B, Wade AR (2006) An Oculomotor Decision Process Revealed by Functional Magnetic Resonance Imaging. 26:13515–13522. https://doi.org/10.1523/JNEUROSCI.4243-06.2006

Henseler I, Krüger S, Dechent P, Gruber O (2011) A gateway system in rostral PFC? Evidence from biasing attention to perceptual information and internal representations. Neuroimage 56:1666–1676. https://doi.org/10.1016/j.neuroimage.2011.02.056

Hubl D, Nyffeler T, Wurtz P, Chaves S (2008) Time course of blood oxygenation level–dependent signal response after theta burst transcranial magnetic stimulation of the frontal eye field. Neuroscience 151:921–928. https://doi.org/10.1016/j.neuroscience.2007.10.049

Ikkai A, Curtis CE (2008) Cortical activity time locked to the shift and maintenance of spatial attention. Cereb Cortex 18:1384–1394. https://doi.org/10.1093/cercor/bhm171

Jamadar SD, Johnson BP, Clough M, et al (2015) Behavioral and neural plasticity of ocular motor control: Changes in performance and fMRI activity following antisaccade training. Front Hum Neurosci 9:1–13. https://doi.org/10.3389/fnhum.2015.00653

Jarvstad A, Gilchrist ID (2019) Cognitive control of saccadic selection and inhibition from within the core cortical saccadic network. J Neurosci 39:2497–2508. https://doi.org/10.1523/JNEUROSCI.1419-18.2018

Kastner S, DeSimone K, Konen CS, et al (2007) Topographic maps in human frontal cortex revealed in memory-guided saccade and spatial working-memory tasks. J Neurophysiol 97:3494–3507. https://doi.org/10.1152/jn.00010.2007

Kim C, Johnson NF, Gold BT (2012) Common and distinct neural mechanisms of attentional switching and response conflict. Brain Res 1469:92–102. https://doi.org/10.1016/j.brainres.2012.06.013

Kurata J, Thulborn KR, Firestone LL (2005) The cross-modal interaction between pain-related and saccade-related cerebral activation: A preliminary study by event-related functional magnetic resonance imaging. Anesth Analg 101:449–456. https://doi.org/10.1213/01.ANE.0000158468.84424.5D

Laird AR, Robinson JL, McMillan KM, et al (2010) Comparison of the disparity between Talairach and MNI coordinates in functional neuroimaging data: Validation of the Lancaster transform. Neuroimage 51:677–683. https://doi.org/10.1016/j.neuroimage.2010.02.048

Lancaster JL, Tordesillas-Gutiérrez D, Martinez M, et al (2007) Bias between MNI and talairach coordinates analyzed using the ICBM-152 brain template. Hum Brain Mapp 28:1194–1205. https://doi.org/10.1002/hbm.20345

Levy I, Schluppeck D, Heeger DJ, Glimcher PW (2007) Specificity of human cortical areas for reaches and saccades. J Neurosci 27:4687–4696. https://doi.org/10.1523/JNEUROSCI.0459-07.2007

Levy BJ, Wagner AD (2011) Cognitive control and right ventrolateral prefrontal cortex: Reflexive reorienting, motor inhibition, and action updating. Ann N Y Acad Sci 1224:40–62. https://doi.org/10.1111/j.1749-6632.2011.05958.x

Lin H, Li WP, Carlson S (2019) A privileged working memory state and potential top-down modulation for faces, not scenes. Front Hum Neurosci 13:1–10. https://doi.org/10.3389/fnhum.2019.00002

Manoach DS, Thakkar KN, Cain MS, et al (2007) Neural activity is modulated by trial history: A functional magnetic resonance imaging study of the effects of a previous antisaccade. J Neurosci 27:1791–1798. https://doi.org/10.1523/JNEUROSCI.3662-06.2007

Mao L, Zhou B, Zhou W, Han S (2007) Neural correlates of covert orienting of visual spatial attention along vertical and horizontal dimensions. Brain Res 1136:142–153. https://doi.org/10.1016/j.brainres.2006.12.031

Melcher T, Gruber O (2006) Oddball and incongruity effects during Stroop task performance: A comparative fMRI study on selective attention. Brain Res 1121:136–149. https://doi.org/10.1016/j.brainres.2006.08.120

Mohanty A, Egner T, Monti JM, Mesulam MM (2009) Search for a threatening target triggers limbic guidance of spatial attention. J Neurosci 29:10563–10572. https://doi.org/10.1523/JNEUROSCI.1170-09.2009

Neggers SFW, Huijbers W, Vrijlandt CM, et al (2007) TMS Pulses on the Frontal Eye Fields Break Coupling Between Visuospatial Attention and Eye Movements. J Neurophysiol 98:2765–2778. https://doi.org/10.1152/jn.00357.2007

Neggers SFW, van Diepen RM, Zandbelt BB, et al (2012) A functional and structural investigation of the human fronto-basal volitional saccade network. PLoS One 7:. https://doi.org/10.1371/journal.pone.0029517

Pierce JE, McDowell JE (2015) Modulation of cognitive control levels via manipulation of saccade trial-type probability assessed with event-related BOLD fMRI. J Neurophysiol 115:763–772. https://doi.org/10.1152/jn.00776.2015

Pierce JE, McDowell JE (2017) Contextual effects on cognitive control and BOLD activation in single versus mixed saccade tasks. Brain Cogn 115:12–20. https://doi.org/10.1016/j.bandc.2017.03.003

Pierce JE, Saj A, Vuilleumier P (2019) Differential parietal activations for spatial remapping and saccadic control in a visual memory task. Neuropsychologia 131:129–138. https://doi.org/10.1016/j.neuropsychologia.2019.05.010

Roth JK, Serences JT, Courtney SM (2006) Neural system for controlling the contents of object working memory in humans. Cereb Cortex 16:1595–1603. https://doi.org/10.1093/cercor/bhj096

Salvia E, Harvey M, Nazarian B, Grosbras MH (2020) Social perception drives eye-movement related brain activity: Evidence from pro- and anti-saccades to faces. Neuropsychologia 139:107360. https://doi.org/10.1016/j.neuropsychologia.2020.107360

Schon K, Tinaz S, Somers DC, Stern CE (2008) Delayed match to object or place: An event-related fMRI study of short-term stimulus maintenance and the role of stimulus pre-exposure. Neuroimage 39:857–872. https://doi.org/10.1016/j.neuroimage.2007.09.023

Sreenivasan KK, Gratton C, Vytlacil J, D'Esposito M (2014) Evidence for working memory storage operations in perceptual cortex. Cogn Affect Behav Neurosci 14:117–128. https://doi.org/10.3758/s13415-013-0246-7

Stelzel C, Basten U, Fiebach CJ (2011) Functional connectivity separates switching operations in the posterior lateral frontal cortex. J Cogn Neurosci 23:3529–3539. https://doi.org/10.1162/jocn_a_00062

Tamber-Rosenau BJ, Asplund CL, Marois R (2018) Functional dissociation of the inferior frontal junction from the dorsal attention network in top-down attentional control. J Neurophysiol 120:2498–2512. https://doi.org/10.1152/jn.00506

Tark KJ, Curtis CE (2009) Persistent neural activity in the human frontal cortex when maintaining space that is off the map. Nat Neurosci 12:1463–1468. https://doi.org/10.1038/nn.2406

Tibber M, Saygin AP, Grant S, et al (2010) The neural correlates of visuospatial perceptual and oculomotor extrapolation. PLoS One 5:1–12. https://doi.org/10.1371/journal.pone.0009664

Todd JJ, Han SW, Harrison S, Marois R (2011) The neural correlates of visual working memory encoding: A time-resolved fMRI study. Neuropsychologia 49:1527–1536. https://doi.org/10.1016/j.neuropsychologia.2011.01.040

Tu PC, Yang TH, Kuo WJ, et al (2006) Neural correlates of antisaccade deficits in schizophrenia, an fMRI study. J Psychiatr Res 40:606–612. https://doi.org/10.1016/j.jpsychires.2006.05.012

Vossel S, Weidner R, Driver J, et al (2012) Deconstructing the architecture of dorsal and ventral attention systems with dynamic causal modeling. J Neurosci 32:10637–10648. https://doi.org/10.1523/JNEUROSCI.0414-12.2012

Wen X, Yao L, Liu Y, Ding M (2012) Causal interactions in attention networks predict behavioral performance. J Neurosci 32:1284–1292. https://doi.org/10.1523/JNEUROSCI.2817-11.2012

Wills KM, Liu J, Hakun J, et al (2017) Neural Mechanisms for the Benefits of Stimulus-Driven Attention. Cereb Cortex 27:5294–5302. https://doi.org/10.1093/cercor/bhw308

Xuan B, Mackie MA, Spagna A, et al (2016) The activation of interactive attentional networks. Neuroimage 129:308–319. https://doi.org/10.1016/j.neuroimage.2016.01.017

Yin S, Deák G, Chen A (2018) Coactivation of cognitive control networks during task switching. Neuropsychology 32:31–39. https://doi.org/10.1037/neu0000406

Zanto TP, Rubens MT, Bollinger J, Gazzaley A (2010) Top-down modulation of visual feature processing: The role of the inferior frontal junction. Neuroimage 53:736–745. https://doi.org/10.1016/j.neuroimage.2010.06.012

Zanto TP, Rubens MT, Thangavel A, Gazzaley A (2011) Causal role of the prefrontal cortex in top-down modulation of visual processing and working memory. Nat Neurosci 14:656–663. https://doi.org/10.1038/nn.2773

Zhang X, Mlynaryk N, Ahmed S, et al (2018) The role of inferior frontal junction in controlling the spatially global effect of feature-based attention in human visual areas. PLoS Biol 16:1–28. https://doi.org/10.1371/journal.pbio.2005399

Zhao Y, Kuai S, Zanto TP, Ku Y (2020) Neural Correlates Underlying the Precision of Visual Working Memory. Neuroscience 425:301–311.https://doi.org/10.1016/j.neuroscience.2019.11.037
